# Traditional Chinese medicine Biqi capsule compared with leflunomide in combination with methotrexate in patients with rheumatoid arthritis: a randomized controlled trial

**DOI:** 10.1186/s13020-020-00319-9

**Published:** 2020-04-23

**Authors:** Zhang Wang, Jiaqi Wu, Dongyun Li, Xuan Tang, Yue Zhao, Xiao Cai, Xianghong Chen, Xiumin Chen, Qingchun Huang, Runyue Huang

**Affiliations:** 1 Institute of Ecological Sciences, School of Life Sciences, South China Normal University, Guangzhou, China; 2 Section Rheumatology Research, The Second Affiliated Hospital of Guangzhou University of Chinese Medicine (Guangdong Provincial Hospital of Chinese Medicine), Guangzhou, China; 3 Guangdong Provincial Key Laboratory of Clinical Research on Traditional Chinese Medicine Syndrome, and State Key Laboratory of Dampness Syndrome of Chinese Medicine, The Second Affiliated Hospital of Guangzhou University of Chinese Medicine, Guangzhou, China

**Keywords:** Rheumatoid arthritis, Biqi capsule, Methotrexate, Leflunomide, Clinical trial, Complementary therapy, Metabolomics, Th2 inflammation

## Abstract

**Background:**

Biqi capsule is a traditional Chinese medicine widely used as a complementary and alternative treatment for rheumatoid arthritis (RA). The objective is to understand the efficacy, safety and mechanism of Biqi combined with methotrexate (MTX) in RA.

**Methods:**

We present a randomized, controlled pilot trial on Biqi combined with MTX against patients with active RA. Seventy patients were randomized 1:1 to receive Biqi + MTX or Leflunomide (LEF) + MTX for 24 weeks, and were assessed at baseline, 4, 12 and 24 weeks. Serum and urine samples were collected for metabolomics.

**Results:**

Overall, 81.2% patients in Biqi group achieved ACR20 at 24 weeks. No statistically significant differences were observed in primary or secondary outcomes between the two groups. A better safety profile was observed for Biqi with significantly fewer adverse effects reported (11.4%) compared to LEF group (40%, *P *< 0.05). Comparison between treatment responders and non-responders indicated a unique urine metabolic profile of enriched fatty acids and decreased acylcarnitines associated with Biqi responders, indicating a restored energy homeostasis in response to Biqi. The gene targets of these metabolites were significantly enriched in interleukin-4 and interleukin-13 pathways, implying that Biqi could ameliorate Th2-derived inflammatory response. Multivariate network analysis indicated that patient morning stiffness and SJC were key factors associated with metabolomics in Biqi-treated patients, whereas CRP was the main factor in LEF group. Therefore, Biqi and LEF likely work by influencing different patient clinical parameters.

**Conclusions:**

Our study suggests that Biqi capsule can be a promising alternative option in combination with MTX for RA treatment, and demonstrates the capability of using metabolomics to interrogate mechanism of action for traditional Chinese medicine.

*Trial registration* This trial is registered with ChiCTR, No. ChiCTR-IPR-16009029. Registered August 15, 2016. http://www.chictr.org.cn/showprojen.aspx?proj=15034

## Background

Rheumatoid arthritis (RA) is an autoimmune disease characterized by progressive articular destruction and associated comorbidities in vascular, metabolic, bone, and psychological domains [1, 2]. Disease-modifying antirheumatic drugs (DMARDs) are recommended by the American College of Rheumatology (ACR) for the treatment of RA [3]. One of the most widely used DMARDs, Methotrexate (MTX) is the anchor drug for treatment of RA [4]. However, some patients failed to achieve low disease activity or clinical remission state after receiving MTX monotherapy [5, 6]. Leflunomide (LEF) is another DMARD with different mode of action from MTX [7]. It was considered as one of the fundamental therapeutics for RA by the European League Against Rheumatism (EULAR) in 2010 [3]. The combination of LEF and MTX (MTX + LEF) has been widely applied for treatment of refractory RA [8]. While it shows improved efficacy, there are also increasing risks of hepatotoxicity, bone marrow suppression, tuberculosis and infection [9–12], which has led an increasing number of RA patients to seek complementary and integrative medicine [13].

Traditional Chinese medicine (TCM) has been practiced in China for thousands of years and it offers a holistic approach to patient management. A variety of TCMs have been used for the treatment of RA in China for centuries, and their efficacy and safety were inferred from clinical experience and tested in clinical trials [14, 15]. Biqi capsule is a well-recognized traditional TCM formula that has been approved by the State Food and Drug Administration as an add-on therapy for RA due to its clinical benefits and low side effects [16]. Biqi capsule consists of ten TCM medicines: Strychni Semen (Maqianzi), Pheretima (Dilong), Codonopsis Radix (Dangshen), Poria (Fuling), Atractylodis Macrocephalae Rhizoma (Baizhu), Glycyrrhizae Radix Et Rhizoma (Gancao), Chuanxiong Rhizoma (Chuangxiong), Salviae Miltiorrhizae Radix Et Rhizoma (Danshen), Notoginseng Radix Et Rhizoma (Sanqi) and Cyathulae Radix (Chuanniuxi). Previous studies showed that Biqi capsule improved clinical symptoms and reduced inflammation in RA patients [17, 18]. A meta-analysis of ten randomized controlled trials (RCTs) revealed that combination treatment with MTX and Biqi (MTX + Biqi) was more effective than MTX monotherapy for RA. However, the majority of the included RCTs showed low methodological quality [19]. Despite its efficacy, little is known regarding the underlying mechanism of action of Biqi capsule for RA due to the complexity of its bioactive ingredients and paucity of knowledge on their therapeutic targets in human body.

Previously, we have explored potential mechanisms of action (MoA) for TCMs in treating RA and other rheumatic disorders through a number of pre-clinical studies including in silico network modeling [20], in vitro cellular assays [20, 21] and in vivo animal experiment [22]. Although insightful, results of these studies warrant further validation in the clinical settings. On the other hand, while a few RCTs have been conducted to assess the efficacy of TCMs in RA [23], a comprehensive understanding of MoA for TCMs by directly measuring the metabolic perturbation and immune response of patients has been lacking. Metabolomics emerge as a useful tool to provide novel insights into the therapeutic effects and underlying mechanisms of multi-biochemical component medicines. In addition, it endows the power to identify biomarkers indicative of treatment response. Therefore, a systems biology approach integrating measurements of clinical parameters and metabolomics (known as pharmacometabolomics [24]) in the RCTs offers the promise to interrogate MoA for TCMs in RA and identify novel biomarkers toward precision medicine.

The aim of this randomized, controlled pilot trial is to evaluate the efficacy and safety of MTX + Biqi compared to MTX + LEF for RA. We also employed a systems biology approach by exploring the differential MoAs for biqi and LEF in treating RA through collective serum and urine metabolomics. Results in this study will serve as a basis for future large-scale studies on this combination therapy and other TCMs in general.

## Methods

### Trial design

This study was a multi-centric, open-label, randomized trial conducted in four hospitals in Guangdong province in China, the Second Affiliated Hospital of Guangzhou University of Chinese Medicine, Shenzhen Hospital of Traditional Chinese Medicine, Dongguan Hospital of Traditional Chinese Medicine and Guangzhou Hospital of Integrated Traditional Chinese and Western Medicine, between September 2016 and July 2018. The study was conducted in accordance with the Declaration of Helsinki. All participants provided written informed consent and the protocol was approved by the Medical Ethics Committee of the Second Affiliated Hospital of Guangzhou University of Chinese Medicine (B2016-073-01) and registered with the World Health Organization clinical trial registry (No. ChiCTR-IPR-16009029).

### Setting and participants

Eligible patients had to meet the following criteria for this trial: (1) aged 18–65 years; (2) RA was confirmed by 2010 ACR/EULAR classification criteria [25]; (3) imaging results suggested Class I, II or III disease (according to the 1987 American Rheumatism Association (ARA) classification standard [26]); (4) Chinese medicine inclusion criteria: according to the syndrome of wind and damp stagnation syndrome, or cold and damp stagnation, or phlegm and stasis stagnation, or deficiency of Qi and Blood [27]; (5) provided written informed consent.

Patients were excluded from the study if they: (1) used in the last month or are using glucocorticoids, MTX, hydroxychloroquine, willow nitrogen sulfanilamide pyridine, cyclophosphamide, penicillamine and gold preparations and other immunosuppressive drugs or slow-acting drugs; (2) had a history of cardiac, hepatic, renal, or mental diseases, other rheumatic autoimmune diseases, any current infection, or any cancer; (3) currently in pregnancy, or were planning on being pregnant during the study period; (4) were unwilling or unable to comply with treatment or assessment regimen; (5) experienced an allergic reaction to the medicine; (6) complicated with active gastrointestinal diseases or diagnosed with esophagus or digestive ulcer in the last month; (7) was participating in another clinical trial within 4 weeks prior to screening.

### Randomization and interventions

Patients were randomly assigned 1:1 to receive either Biqi capsule at a dose of 1.2 g twice daily, or LEF at a dose of 20 mg once daily, for a period of 24 weeks. All included patients received MTX at a dose of 10–15 mg once weekly with 5 mg of folic acid two or three times a day. Participants were allowed to take glucocorticoids at 2.5–10 mg/day if their patient’s assessment of pain were more than 40 mm on a 100-mm visual analogue scale. In addition, nonsteroidal anti-inflammatory drugs (NSAIDs), calcitriol/calcium carbonate and antacids were allowed during the study. Patients could discontinue the assigned treatment at any time for lack of effectiveness or adverse effects or by their own choice.

Randomization was performed by an independent statistician using SAS v9.2. Allocation concealment was achieved using a centralized service. The statistician who prepared the list had no further role in the study. We did not use stratification or blocking. Blinding was not practical for this investigator-initiated clinical trial, and the treatments were open to both the researchers and participants.

### Outcomes and measurements

Outcomes were assessed at baseline and after 4, 12 and 24 weeks. The primary efficacy endpoint was the proportion of patients who achieved a 20% improvement in ACR criteria (ACR20) at 24 weeks, defined as at least a 20% reduction from baseline in the number of both tender and swollen joints (TJC and SJC) and at least a 20% improvement in three or more of the following: the physician’s or patient’s global assessment of disease activity (PhGADA or PaGADA), the patient’s assessment of pain on a visual analogue scale (VAS), the patient’s assessment of function using the Health Assessment Questionnaire (HAQ), and the serum erythrocyte sedimentation rate (ESR) or C-reactive protein (CRP) level [28]. Secondary endpoints included the ACR50 and ACR70, EULAR response, and other clinical parameters (supplementary methods). The EULAR response criteria were assessed based on the 28-joint disease activity score (DAS28). A moderate EULAR response was a decrease of > 0.6 and ≤ 1.2, and a good response is a decrease of > 1.2 [29]. All of the outcome measures were assessed and recorded at baseline, weeks 4, 12 and 24 by trained evaluators who were unaware of the specific therapeutic regimen.

Safety endpoints included adverse events (AEs), serious AEs, and laboratory abnormalities. Safety was monitored by physical examination, chest radiography, electrocardiography, blood pressure, pulse rate and body temperature. Standard hematological and biochemical tests and urinalysis were also performed. The occurrence of adverse events was documented and included those spontaneously reported by patients, as well as responses elicited by general questioning.

### Statistical analysis

The sample size was calculated based on the primary outcome ACR20 [30, 31]. It was estimated that 98 participants (49 per arm) is sufficient to detect a 20% difference in ACR20, using a 5% significance level with 80% power from baseline to end of treatment, allowing for a 20% loss to follow-up. Full analysis set was used to test differences in baseline characteristics of patients between two groups. All efficacy endpoints of the two group were analyzed using the intention-to-treat (ITT) and per-protocol set (PP). The ITT analysis included all patients who received at least one dose of the drug, and was performed as the primary outcome analysis. The PP analysis included patients who strictly followed the protocol and completed the study at each time point.

The mean ± standard deviation were used for the description of continuous variables. The proportion was used for the description of categorical variables. Dichotomous variables were analyzed using Fisher’s exact test for the primary efficacy endpoints (ACR), the EULAR response, as well as the safety endpoints. The secondary efficacy endpoints, including the TJC28, SJC28, patient’s assessment of pain, PaGADA, PhGADA, CRP and ESR, were assessed by one-way repeated measures ANOVA of the mean values from baseline to weeks 4, 12 and 24 for each group, followed by Benjamin-Hochberg (BH) post hoc correction to address the multiple comparisons on clinical measures and in between timepoints. The differences between the two treatment groups in these secondary efficacy endpoints were analyzed by the non-parametric Mann–Whitney U test to accommodate their non-normal distributions. All statistical tests were 2-sided. *P*-values less than 0.05 were considered significant. An independent statistician who was blinded to the group allocation conducted the data analysis using SPSS software (version 17.0).

### Serum and urine metabolomic analysis

Serum and urine samples were collected from patients at baseline, weeks 4, 12 and 24, and were analyzed using Agilent 1290 ultra-performance liquid chromatography mass spectrometry (UHPLC-MS). Identification of metabolites was performed by searching HMDB [32], METLIN [33] and KEGG databases [34]. Metabolomic data analysis was performed using MetaboAnalyst [35]. The data were normalized by sum, log2 transformed and auto-scaled. Analysis of similarities (ANOSIM) was performed to test the dissimilarity between metabolic profiles among groups. Differentially abundant metabolites were identified for paired samples in between different timepoints using one-way repeated measures ANOVA. Differential metabolites at 24 weeks between treatment responders and non-responders were identified using analysis of covariance (ANCOVA), in which the metabolite abundances at baseline were controlled as a covariate. Human gene targets of the metabolites were identified using the STITCH database [36]. Metabolite-target interactions with activation or inhibition effects and with confidence score > 0.8 were retained. The fold-changes of targets were inferred as the additive fold-change of all metabolites targeting the genes, adjusted by activation or inhibition effects of the metabolites to the genes.

Correlation was performed between serum and urine metabolites and patient clinical variables. Each metabolite and clinical variable was first residualized using a linear mixed-effect model with age, gender and timepoint as fixed-effect variables and patient identity as random-effect variable. A hierarchical spearman correlation was performed on the residues of metabolites and clinical variables using HAllA [37]. Correlation networks were established using the top 50 correlations between metabolites and clinical variables.

## Results

### Patient characteristics

**Fig. 1:**
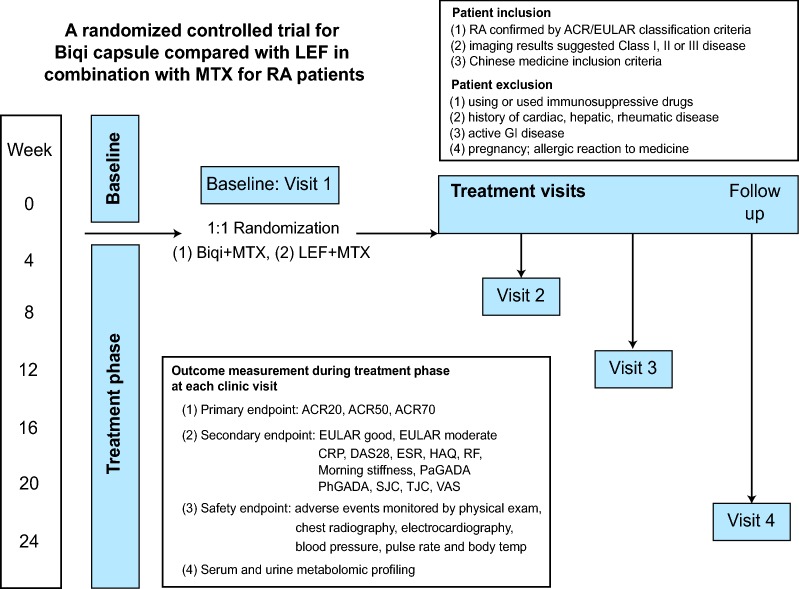
The main procedure for the clinical trial

**Fig. 2:**
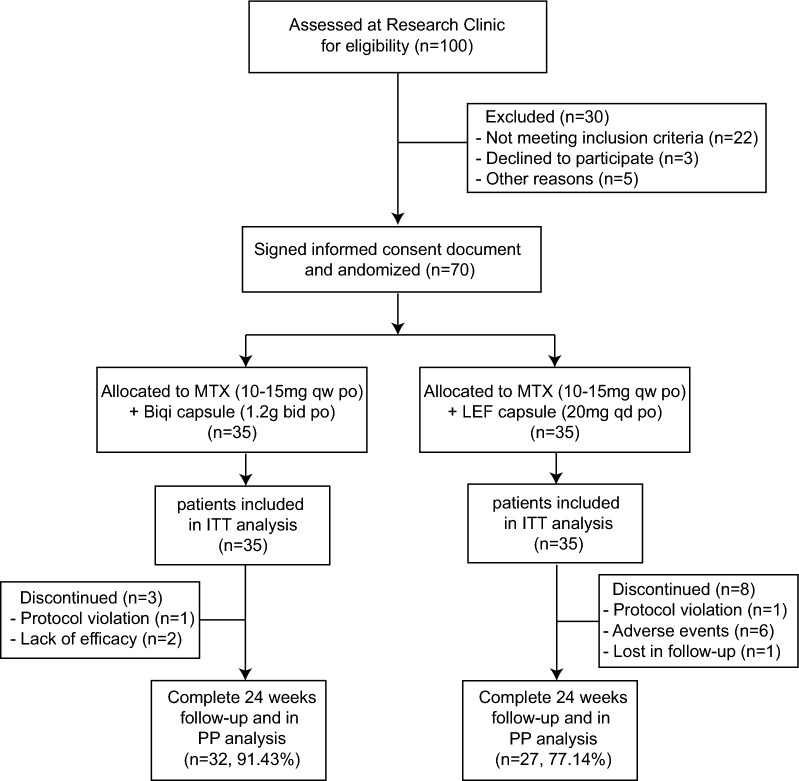
Consort diagram for patient participation flow throughout the trial

The main procedure of the clinical trial was illustrated in Fig. 1. Of 100 screened patients, 70 eligible patients were enrolled in the study and 1:1 randomly assigned to one of the two treatment arms (Fig. 2). Fifty-nine (84.3%) patients completed 24 weeks of evaluation (32 in Biqi and 27 in LEF group). There were no statistically significant differences between two groups in demographics, disease characteristics or concomitant medications at baseline (Table 1, Additional file 1: Table S1).

**Table 1:** The demographic and clinical characteristics of RA patients at baseline in the full analysis set

Characteristics	MTX + Biqi (n = 35)	MTX + LEF (n = 35)	*P* value
Age (SD), years	42.74 (10.35)	44.40 (11.36)	0.526
Female, n (%)	32 (91.43)	31 (88.57)	0.690
Disease duration (SD), months	26.12 (31.46)	35.19 (33.45)	0.136
TJC (SD), n	8.97 (6.31)	8.29 (6.42)	0.544
SJC (SD), n	6.03 (5.34)	4.23 (3.03)	0.246
Patient’s assessment of pain (SD), mm	58.57 (24.54)	56.29 (21.43)	0.678
PhGADA^†^(SD), mm	52.09 (25.53)	52.00 (20.37)	1.000
PaGADA^†^(SD), mm	55.71 (25.61)	53.00 (20.37)	0.673
Morning stiffness (SD), min	69.00 (53.99)	56.03 (49.06)	0.365
HAQ, mean ± SD	1.02 (0.81)	0.73 (0.73)	0.116
hs-CRP (SD), mg/L	12.45 (18.76)	15.88 (17.27)	0.259
ESR (SD), mm/h	44.49 (27.20)	54.86 (27.13)	0.115
RF^#^ (SD), U/mL	153.55 (162.72)	174.33 (183.97)	0.787
Anti-CCP^#^, positive rate	28 (84.85)	29 (82.86)	1.000
DAS28 (SD)	4.66 (1.39)	4.55 (1.22)	0.742
NSAIDs, n (%)	29 (82.9%)	28 (80.0%)	0.759
Glucocorticoid oral, n (%)	6 (20.0%)	14 (37.1%)	0.112
Folic acid tablet, n (%)	23 (65.7%)	27 (77.1%)	0.290
Antacids, n (%)	27 (77.1%)	26 (74.3%)	0.780

Data are presented as the mean (SD) or n (%)

*TJC* tender joint count, *SJC* swollen joint count, *PaGADA* patient’s global assessment of disease activity, *PhGADA* physician’s global assessment of disease activity, *HAQ* Health Assessment Questionnaire, *ESR* erythrocyte sedimentation rate, *CRP* C-reactive protein, *RF* rheumatoid factor, *anti-CCP* anti-cyclic citrullinated peptide antibody, *DAS28* 28-joint disease activity score, *cDAI* clinical disease activity index

RF^#^ was measured by immunonephelometry with a cut-off value of 20 U/mL. Anti-CCP^#^ was measured using a commercially available second-generation ELISA kit (Abbott, USA) with a cut-off value of 25 U/mL

^†^Measured on a 100-mm visual analog scale. NSAID: nonsteroidal anti-inflammatory drug

### Comparable clinical efficacy for Biqi and LEF treatment groups

**Fig. 3:**
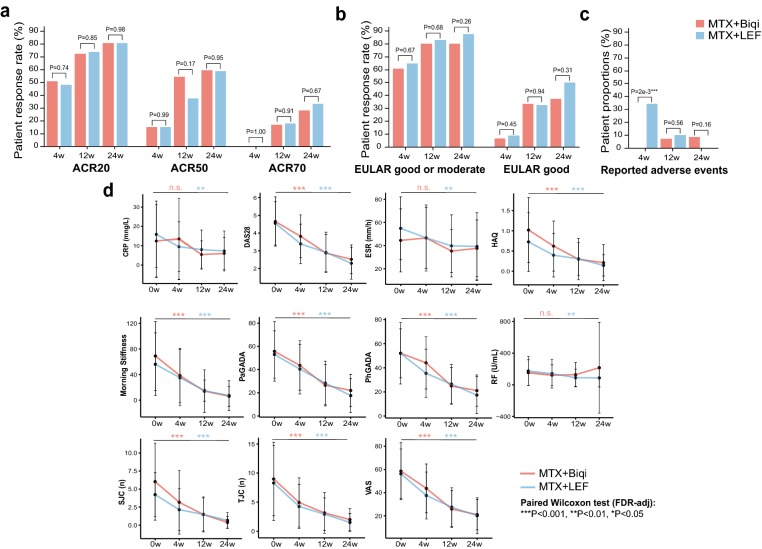
Patient clinical outcomes for Biqi and LEF group in the ITT analysis. These include **a** patient response rates for ACR20, ACR50, ACR70, **b** patient response rates for EULAR good or moderate response, **c** proportion of patients with reported adverse events, and **d** patient clinical measurements at baseline, 4, 12 and 24 weeks. Paired Wilcoxon test was performed followed by Benjamin-Hochberg (BH) post hoc correction for multiple comparisons on clinical measures and in between timepoints. The FDR-adjusted *P*-values between 24 weeks and baseline were reported. *** FDR *P *< 0.001, ** *P *< 0.01, * *P *< 0.05

Overall similar clinical efficacy was observed for the two treatment arms. 81.2% of patients in Biqi group and 81.5% of patients in LEF group achieved ACR20 response as the primary outcome at 24 weeks (Fig. 3a). Similar ACR50 and ACR70 rates were observed at 24 weeks for the two groups. The ACR20, ACR50 and ACR70 rates were also comparable between two groups during 4 and 12 weeks, except for a notable higher ACR50 rate for Biqi compared to LEF group at 12 weeks (51.5% versus 35.4%, *P *= 0.17). There were slightly higher patient EULAR response rates in LEF compared to Biqi group (Fig. 3b). Compared to baseline, most clinical measurements showed significant and continuous improvement in both two groups over time (Fig. 3d, FDR-adjusted *P *< 0.05). No significant differences were found between the two groups. PP analysis on the 59 patients that completed 24 weeks of treatment showed consistent results with those in the ITT analysis (Additional file 1: Fig. S1).

### A better safety profile for Biqi treatment

Of all 70 patients, 18 (25.7%) experienced one or more adverse events (AEs), including 4 (11.4%) and 14 (40%) patients receiving Biqi and LEF, respectively (Fig. 3c, *P *= 0.006). In Biqi group, all AEs were regarding to elevated liver enzymes above the normal range. In LEF group, the AEs included hepatic side effects (9 cases), abdominal discomfort (2 cases), hypertension (1 case), rash (1 case), and herpes zoster (1 case). In particular, 5 patients in LEF group suffered from the increasing ALT/AST and discontinued the study. Moreover, 12 of the 14 LEF-treated patients with AEs showed symptoms at week 0 or week 4, whereas all AEs were for Biqi-treated patients were observed at week 12 or 24 (week 4: *P *= 2e−3, Fig. 3c). These results indicate a milder adverse effect of Biqi possibly due to its slow-acting nature, as opposed to LEF which likely provoked an acute host response at the onset of treatment.

### Serum and urine metabolomics over time for Biqi and LEF treatment arms

**Fig. 4:**
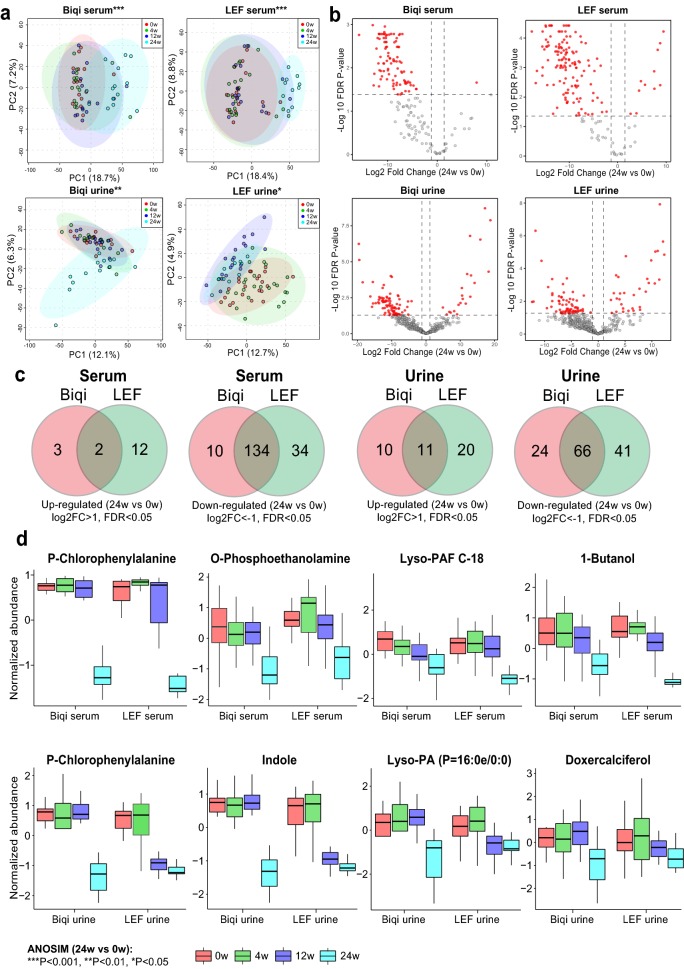
Serum and urine metabolic shifts over time for Biqi and LEF groups. **a** PCA plots for serum and urine samples at baseline, 4, 12 and 24 weeks for patients receiving Biqi or LEF treatment respectively. Samples were colored by timepoints. The ellipses represent 95% confidence interval for samples within each timepoint. **b** Volcano plots showing differentially abundant serum and urine metabolites comparing 24 weeks versus baseline for Biqi and LEF-treated patients. **c** Venn diagrams comparing serum and urine metabolites up and down-regulated at 24 weeks versus baseline for Biqi and LEF groups. **d** Top serum and urine metabolites that were commonly downregulated in Biqi and LEF groups

To investigate potential mechanism of action for Biqi capsule, we carried out metabolomic analysis for patient serum and urine samples. A total of 106 serum and 103 urine samples were collected from 31 to 27 patients respectively (Additional file 1: Table S2). Quality assessment indicated that all samples had good repeatability and stability (Additional file 1: Fig. S2). A total of 14,956 serum and 18,775 urine metabolites were detected in UHPLC-MS, of which 275 and 474 metabolites were successfully resolved to identity. There were significant serum metabolomic shifts at 24 weeks in both Biqi and LEF groups as indicated in PCA plots (ANOSIM 24w vs baseline: *P *< 0.05, Fig. 4a, b). Similar trend was observed for urine samples of Biqi-treated patients, whereas for LEF-treated patients both week 12 and 24 samples together formed a separate cluster from other samples. Consistently, the majority of differentially abundant serum and urine metabolites were identified at 24 weeks for Biqi-treated patients, while for LEF-treated patients the number of differential urine metabolites peaked at 12 weeks (Additional file 1: Fig. S3). The different timing for patient metabolomic shifts supported the slow-acting nature of Biqi compared to LEF.

Next, we identified differentially abundant metabolites at 24 weeks versus baseline for Biqi and LEF groups. Comparison of the differential metabolites indicates a large proportion of serum and urine metabolites commonly altered in both groups. For example, 136 out of 195 (69.7%) differential serum metabolites were commonly altered in both Biqi and LEF groups at 24 weeks (FDR P < 0.05, log2 fold-change (FC) > 1). Of them, 134 metabolites were downregulated (Fig. 4c, Additional file 1: Table S3). Likewise, 77 urine metabolites were commonly altered in both groups, of which 66 metabolites were downregulated (Fig. 4c, Additional file 1: Table S3). Amino acid and sphingolipid metabolisms were most significantly enriched for the downregulated metabolites in both serum and urine (Fig. Additional file 1: S4a, b). P-Chlorophenylalanine was most significantly decreased in serum and urine for both groups (Fig. 4d). Of note, for the metabolites that were deemed uniquely up or downregulated in each treatment group, they mostly showed similar trend of changes in the other group, despite at a smaller scale that didn’t reach statistical significance (Additional file 1: Fig. S5). Therefore, overall patients in Biqi and LEF groups appeared to have similar serum and urine metabolomic shifts over time.

### Urine metabolomic profiles associated with patient responsiveness to Biqi treatment

**Fig. 5:**
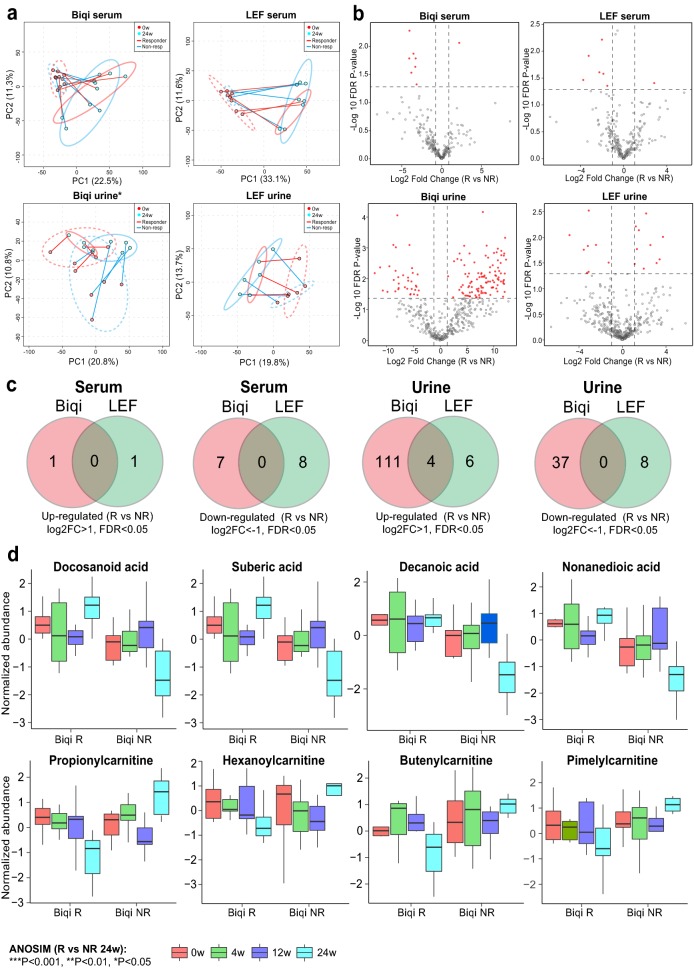
Serum and urine metabolomic profiles for responders and non-responders to Biqi and LEF treatment. **a** PCA plots for paired serum and urine samples at baseline and 24 weeks for patients receiving Biqi or LEF treatment respectively. Samples were colored by timepoints. The paired baseline and 24 weeks samples from the same patient were linked by solid lines colored by responders or non-responders. The dotted and solid ellipses represent 95% confidence interval for samples at baseline and 24 weeks, respectively. **b** Volcano plots showing differential abundant serum and urine metabolites comparing responders versus non-responders at 24 weeks in Biqi and LEF groups using ANCOVA. **c** Venn diagrams comparing serum and urine metabolites up and down-regulated in responders versus non-responders at 24 weeks for Biqi and LEF groups. **d** Top urine carboxylic acids and acylcarnitines that were uniquely up and downregulated in Biqi responders versus non-responders at 24 weeks

To further disentangle patient metabolomic shifts as a result of Biqi or LEF treatment alone, we divide both patient groups into responders (R) and non-responders (NR) based on their clinical outcomes at 24 weeks. We chose to use the ACR50 as the criteria for treatment responsiveness which divided patients into two subgroups with roughly even sample size. As expected, PCA indicated significant serum and urine metabolic shifts for both responders and non-responders at week 24 versus baseline for both treatment groups. There was no clear separation between R and NR groups for serum metabolites of both Biqi and LEF-treated patients, and for urine metabolites of LEF-treated patients (Fig. 5a). However, for urine metabolites of Biqi-treated patients, the R and NR were well separated in PCA both at baseline and 24 weeks, indicating a significant association of urine metabolomic profiles with Biqi treatment responsiveness (ANOSIM *P *= 0.039, Fig. 5a, b).

We then used an ANCOVA approach to identify metabolites that significantly differed between R and NR for Biqi and LEF treatments at 24 weeks, controlling for their initial abundances at baseline. A total of 152 urine metabolites were significant different in abundance between Biqi R and NR, 111 uniquely upregulated in Biqi responders (Fig. 5c, Additional file 1: Table S4). In comparison, only 18 metabolites were significantly altered between LEF R and NR at 24 weeks. The top downregulated metabolite in LEF responders was uracil, which was congruent with the established mechanism of action for LEF in inhibiting synthesis of pyrimidine ribonucleotide uridine monophosphate [38]. The upregulated metabolites in Biqi responders were most enriched in propionate and glutathione metabolisms (Additional file 1: Fig. S4c). Meanwhile, multiple medium or long-chain carboxylic acids such as suberic acid, decanoic acid, docosatrienoic acid and hexadecanedioic acid were significantly upregulated (Fig. 5d). The 37 downregulated metabolites in Biqi responders included a wide range of acylcarnitines, including butenylcarnitine, pimelylcarnitine, hexanoycarnitine and propionylcarnitine (Fig. 5d, Additional file 1: Table S4).

To understand how perturbations of metabolites might impact host immune and inflammatory response, we performed an in silico analysis to identify human gene targets for these 152 metabolites using the STITCH database [36]. A total of 167 human gene targets were identified as potentially activated or inhibited by these metabolites (confidence score > 0.8, Additional file 1: Fig. S6). Of them, 91 genes were inferred to be downregulated in Biqi R versus NR at 24 weeks. These genes were most significantly enriched in interleukin (IL)-4 and IL-13 signaling pathways, key Th2-derived anti-inflammatory pathways (Additional file 1: Fig. S7). Conversely, no pathway was significantly enriched for the 70 upregulated genes.

### Distinct associations between metabolites and clinical parameters for Biqi and LEF treatments

**Fig. 6:**
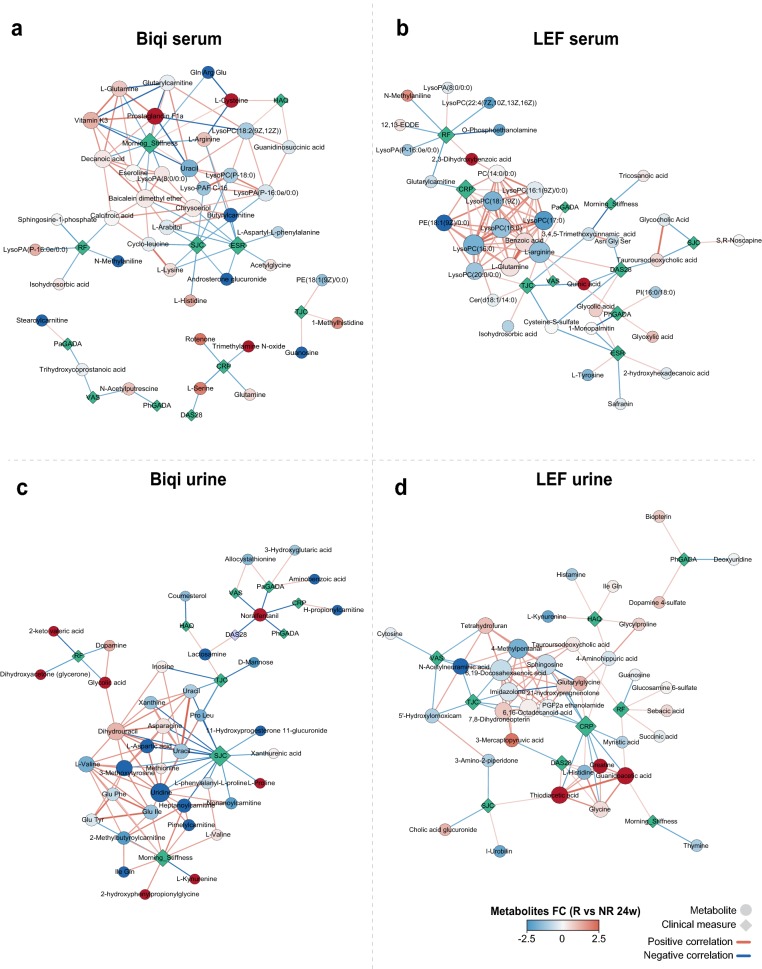
Interaction networks between metabolites and patient clinical parameters in Biqi and Lef group. This includes **a** serum of Biqi-treated patients, **b** serum of LEF-treated patients, **c** urine of Biqi-treated patients, and **d** urine of LEF-treated patients. Each node in round shape represents a metabolite colored based on its fold-change in responders versus non-responders at 24 weeks. Each node in diamond shape represents one clinical measurement. Each edge represents a significant correlation between the two nodes (FDR *P *< 0.05, residual correlation), colored by positive or negative correlations. For visualization purpose, only top 50 significant correlations between metabolites and patient clinical variables, along with any significant correlations within these metabolites are shown

To further link metabolic alternations to patient clinical outcomes, we performed a correlation network analysis between serum and urine metabolites and patient clinical parameters. We used residualized correlation to identify correlations independent of longitudinal measurements and other demographic co-factors. We observed distinct correlation network modules between serum and urine metabolites and clinical variables for Biqi and LEF groups (Fig. 6). For Biqi-treated patients, SJC and morning stiffness were key clinical factors associated with both serum and urine metabolites (Fig. 6a, c). For example, serum l-glutamine, vitamin-D3, decanoic acid and prostaglandin F1a showed significant negative correlations with patient morning stiffness levels (Fig. 6a). Notably, these metabolites were uniquely upregulated in Biqi responders at 24 weeks, suggesting such correlations were also related to Biqi responsiveness. Conversely, multiple urine acylcarnitines, all downregulated in Biqi responders, showed positive correlations with morning stiffness (Fig. 6c). For LEF-treated patients, the main clinical factor associated with metabolites was CRP (Fig. 6b, d). In particular, there was a mutual positive association between multiple serum lysophosphatidylcholines (lysoPCs) that together were positively correlated with CRP (Fig. 6b). Urine metabolites such as ethanolamide and octadecanoid acid were also negatively associated with CRP in LEF group (Fig. 6d).

## Discussion

This 24-week pilot trial confirmed both efficacy and tolerability for Biqi, one of the commonly prescribed TCM formulas, in combination with MTX for RA treatment. Intergroup comparisons suggested an approximately equivalent efficacy of Biqi when compared with LEF that had demonstrated therapeutic efficacy for patients with refractory or active RA [39]. The proportion of patients who attained the primary and secondary endpoints were comparable between LEF and Biqi groups at 4, 12 and 24 weeks. A slightly higher proportion of patients achieved EULAR response in LEF than Biqi group, whereas higher ACR50 was observed for Biqi group in 12 weeks. All clinical measurements were significantly improved at 24 weeks in both groups, except for the non-significant changes in CRP, ESR and rheumatoid factor (RF) in Biqi group. Together these results indicated a clear clinical efficacy for MTX + Biqi comparable to MTX + LEF combination therapy.

Importantly, our results indicated that MTX + Biqi had a better safety profile. The frequency of total adverse events in Biqi group was significantly lower than that of LEF group. Furthermore, all adverse events in Biqi group occurred after 4 weeks, as opposed to those in LEF that occurred at the onset of the treatment, indicating a slow-acting nature for Biqi capsule. Liver abnormality was the most frequently reported adverse events in both treatments. All patients in Biqi group could be immediately relieved after treatment with hematinics, while 14.3% patients in the LEF group discontinued the trial. Reported adverse effects suggested that there were concerns regarding the toxicity of Biqi owing to its active ingredients such as strychnine and brucine [40]. Future studies focused on the possible adverse effects of Biqi in vital organs and systems are therefore highly essential.

The serum and urine metabolomic profiling provided many insights into the potential mechanisms of action for Biqi that might be different from those of LEF. Overall there were similar serum and urine metabolic changes with decreased amino acid and sphingolipid metabolism in both groups when comparing 24 weeks versus baseline. This might be explained as the effect of concomitant MTX treatment that could have masked that of Biqi and LEF. Further patient stratification revealed a unique association of urine metabolomics with Biqi responsiveness, which was characterized by enriched carboxylic acids and decreased acylcarnitines. As acylcarnitines constitute the rate-limiting step for fatty acid beta-oxidation, the increased fatty acids coupled with decreased carnitine together indicates a restored energy homeostasis accompanied with Biqi responsiveness, which may also reflect suppressed host immune responses by Biqi [41]. Accordingly, in silico analysis showed that these metabolites might inhibit cytokines in Th2-derived (IL-4 and IL-13) inflammatory pathways, including IL-1, IL-4, IL-6, IL-8 and IL-13. Bian et al. showed that Biqi inhibited the progression of collagen-induced arthritis by decreasing JAK3, STAT3, IL–4, IL–1 and other inflammatory cytokines in RA rat models [42]. Other clinical studies also confirmed that Biqi markedly decreases IL–17, IL–1 and TNF–a in RA patients [43, 44]. Our results extended these findings in the clinical setting and further supported the anti-inflammatory properties of Biqi capsule.

Using a systems biology approach integrating metabolomics and patient clinical parameters, we showed that there were different types of interactions between metabolites and disease activities in Biqi and LEF groups. In Biqi-treated patients, patient morning stiffness levels were positively correlated with serum anti-inflammatory agents such as prostaglandin F1a [45], vitamin K3 [46] and l-glutamine [47] while negatively correlated with urine acylcarnitines. Importantly, many of these metabolites were also associated with Biqi responsiveness (such as urine acylcarnitines), which further supported that they were key components mediating patient clinical response to Biqi. On the other hand, CRP was the main factor associated with metabolites in the LEF group. The anti-CRP effect of LEF in RA is well established [48] and our interaction network implies that LEF could potentially reduce CRP levels via decreasing lysoPCs in serum, and increasing creatine, guanidinoacetic acid and thiodiacetic acid in urine. Our analysis suggests that Biqi and LEF likely achieved anti-RA effects through impacting different types of patient clinical characteristics. The integrated patient stratification and clinical correlation analyses allowed us to navigate the important metabolites that most likely played mechanistic roles underlying Biqi treatment.

Metabolomics has been increasingly applied to animal experiments and clinical studies on TCMs to characterize their therapeutic efficacies and mechanisms of action in treating RA [49]. For instance, by applying GC–MS based metabolomic approach in a rat model, Wang Y. et al. investigated the effect of Simiaowan and showed that it achieved the anti-arthritic effect by decreasing amino acid metabolism and restoring energy metabolism [50]. Some of their findings were also observed in our results. Comparative studies with additional TCMs in RA are therefore of interest to further determine whether there might be shared or specific metabolic effects between different TCMs in the treatment of RA. In another study, using combined untargeted and targeted metabolomic methods, Wang M. et al. examined the plasma metabolic perturbation in patients receiving a combo therapy of MTX and Chinese herbal medicine tripterygium glycosides, and showed that it modified a wide range of human metabolism including amino acid, nucleic acid, lipid metabolisms and oxidative stress [51]. By comparing the combo-therapy group with mono-therapy groups, they further showed the combo treatment may have specific effects on the levels of alanine, adenosine, and lactic acid. Their and our studies both demonstrated the importance of a rigorous clinical trial design with proper control arm, longitudinal follow-up, and a finer-scale patient stratification analysis, in order to distinguish the metabolic effects specific to TCMs from those non-specific effects from other concomitant treatments or interpersonal variations.

This pilot study has several limitations. Firstly, our study was an open-label clinical trial. In order to achieve more objective outcomes, a double-blind RCT would be necessary. The second one is the duration of the study. According to the EULAR guiding principle for RA treatment, it is usually admitted that a 24 weeks duration study is sufficient to evaluate the statistically significant efficacy of a therapeutic regimen [52]. However, the evaluation of radiological progression of the affected joints in X-ray is not available and requires probably studies of longer duration. The third one is the relatively small cohort size, in particular with respect to safety evaluation and sub-analysis of treatment responsiveness. Future studies with double-blind design, larger group of patients and a prolonged period of time are needed to validate findings of this pilot study and to identify the subgroup of patients best responsive to Biqi capsule.

## Conclusions

Our study showed that Biqi capsule in combination of MTX achieved equivalent efficacy with MTX + LEF in the treatment of RA with a significantly better safety profile. We showed that Biqi capsule might ameliorate RA symptoms through restoring urine energy homeostasis and suppressing Th2 inflammatory response. The improvement in symptoms and patient quality of life makes this combination therapy an attractive alternative treatment to currently available agents for RA patient management. However, the long-term effects and toxicities of Biqi capsule in combination with MTX or other antirheumatic therapies need to be addressed in future studies.

## Supplementary information

**Additional file 1.** Patient clinical outcomes for Biqi and LEF treatment arms in the PP analysis.

## Data Availability

The datasets generated and/or analyzed during the current study are available from the corresponding author on reasonable request.

## Abbreviations

ACR: American College of Rheumatology
ANCOVA: Analysis of covariance
ANOSIM: Analysis of similarities
CRP: C-reactive protein
DAS28: 28-joint disease activity score
DMARDs: Disease-modifying antirheumatic drugs
EULAR: European league against rheumatism
ESR: Erythrocyte sedimentation rate
HAQ: Health Assessment Questionnaire
LEF: Leflunomide
LysoPCs: Lysophosphatidylcholines
ITT: Intention-to-treat
MTX: Methotrexate
PaGADA: Patient’s global assessment of disease activity
PhGADA: Physician’s global assessment of disease activity
PP: Per-protocol
RA: Rheumatoid arthritis
RF: Rheumatoid factor
VAS: Visual analogue scale
