# Supplementary material for: Traditional Chinese medicine Biqi capsule compared with leflunomide in combination with methotrexate in patients with rheumatoid arthritis: a randomized controlled trial
Source: Chin Med. 2020 Apr 23;15:36. doi: 10.1186/s13020-020-00319-9 (PMC7178961; doi:10.1186/s13020-020-00319-9)
Supplement: Supplementary file 1 — Additional file 1. Patient clinical outcomes for Biqi and LEF treatment arms in the PP analysis. [file 13020_2020_319_MOESM1_ESM.docx]

**Traditional Chinese medicine Biqi capsule compared with leflunomide in combination with methotrexate in patients with rheumatoid arthritis: a randomized controlled trial**

Additional file

**Additional methods**

**Participants**

Patients with active RA were recruited from four hospitals in Guangdong China: the Second Affiliated Hospital of Guangzhou University of Chinese Medicine (Guangdong Provincial Hospital of Chinese Medicine), Shenzhen Hospital of Traditional Chinese Medicine, Dongguan Hospital of Traditional Chinese Medicine and Guangzhou Hospital of Integrated Traditional Chinese and Western Medicine.

**Patient inclusion/exclusion criteria**

Eligible patients had to meet the following criteria for this trial: (1) RA was confirmed by 2010 ACR/EULAR classification criteria[1]; (2) imaging results suggested Class I, II or III disease (according to the 1987 American Rheumatism Association (ARA) classification standard[2]); (3) Chinese medicine inclusion criteria: according to the syndrome of wind and damp stagnation syndrome, or cold and damp stagnation, or phlegm and stasis stagnation, or deficiency of Qi and Blood[3]; (4) patients aged between 18 and 65 years; (5) volunteered for the research and signed the informed consent. Patients were excluded from the study if they: (1) used in the last month or using glucocorticoids, MTX, hydroxychloroquine, willow nitrogen sulfanilamide pyridine, cyclophosphamide, penicillamine and gold preparations and other immunosuppressive drugs or slow-acting drugs; (2) had a history of severe, progressive or uncontrolled cardiac, hepatic, renal, or mental diseases, other rheumatic autoimmune diseases, any current infection, or any cancer; (3) currently in pregnancy, or were planning on becoming pregnant during the study period; (4) were unwilling or unable to comply with treatment or assessment regimen; (5) experienced an allergic reaction to the medicine; (6) complicated with active gastrointestinal diseases or diagnosed with esophagus or digestive ulcer in the last month; (7) was participating in another clinical trial within 4 weeks prior to screening.

Participants were allowed to take glucocorticoids at 2.5-10 mg/day if their patient’s assessment of pain were more than 40 mm on a 100-mm visual analogue scale. In addition, nonsteroidal anti-inflammatory drugs (NSAIDs), calcitriol/calcium carbonate and antacids were allowed during the study.

**Patient safety endpoints assessment**

Safety endpoints included adverse events (AEs), serious AEs (SAEs), and laboratory abnormalities. Safety was monitored by physical examination, chest radiography, electrocardiography, blood pressure, pulse rate and body temperature. Standard hematological and biochemical tests and urinalysis were also performed. The occurrence of adverse events was documented and included those spontaneously reported by patients, as well as responses elicited by general questioning.

**Metabolomic analysis**

Methanol (300 μl) and 2-Chloro-L-phenylalanine (20 μl) were added to each serum or urine sample (100 μl). Mixtures were vortexed for 30 sec and then sonicated for 5 min. After standing still for 2 h at -20 degree, mixtures were centrifuged at 13,000 rpm for 15 min. The supernatants (200 μl) were transferred to vitals for liquid chromatographic metabolomic analysis using Agilent 1290 ultra-performance liquid chromatography mass spectrometry (UHPLC-MS) system. Quality control samples were prepared by mixing an equal aliquot (40 μl) from each study sample for the optimization of the UHPLC-MS conditions. Raw data were converted into mzXML format using ProteoWizard and processed by XCMS[4] for peak recognition, alignment, and correction. A visual data matrix containing retention time, m/z pairs, sample names and normalized ion intensities was generated and exported to SIMCA-P 11.5 (Umetrics AB, Umea, Sweden). For other metabolomic and statistical analyses please refer to the main text.


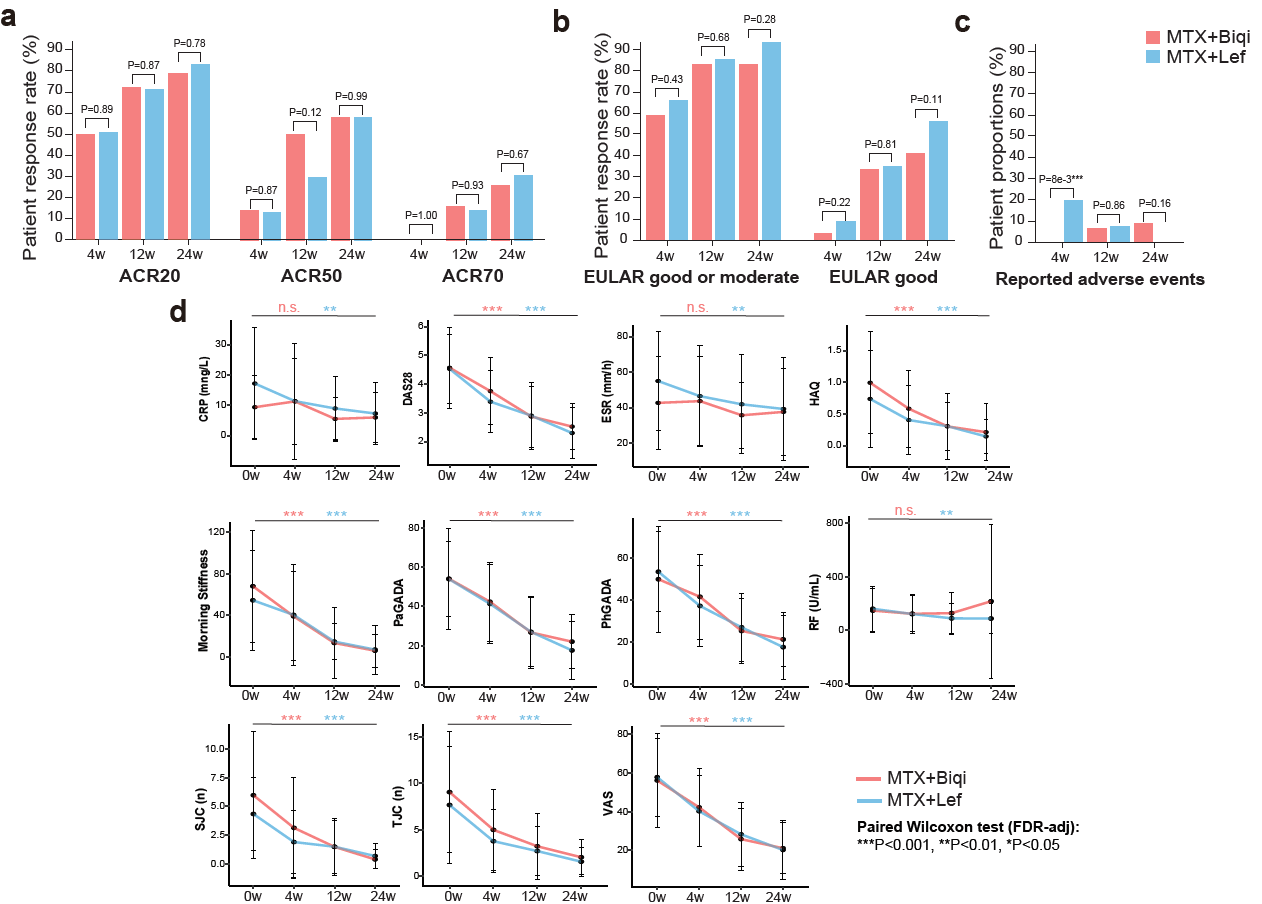


**Fig. S1.** Patient clinical outcomes for Biqi and LEF groups in the PP analysis, in which only patients successfully finished treatment over the entire 24 weeks were included. These include a) patient response rates for ACR20, ACR50, ACR70, b) patient response rates for EULAR good or moderate response, c) proportion of patients with reported adverse events, and d) patient clinical parameters at baseline, 4, 12 and 24 weeks.


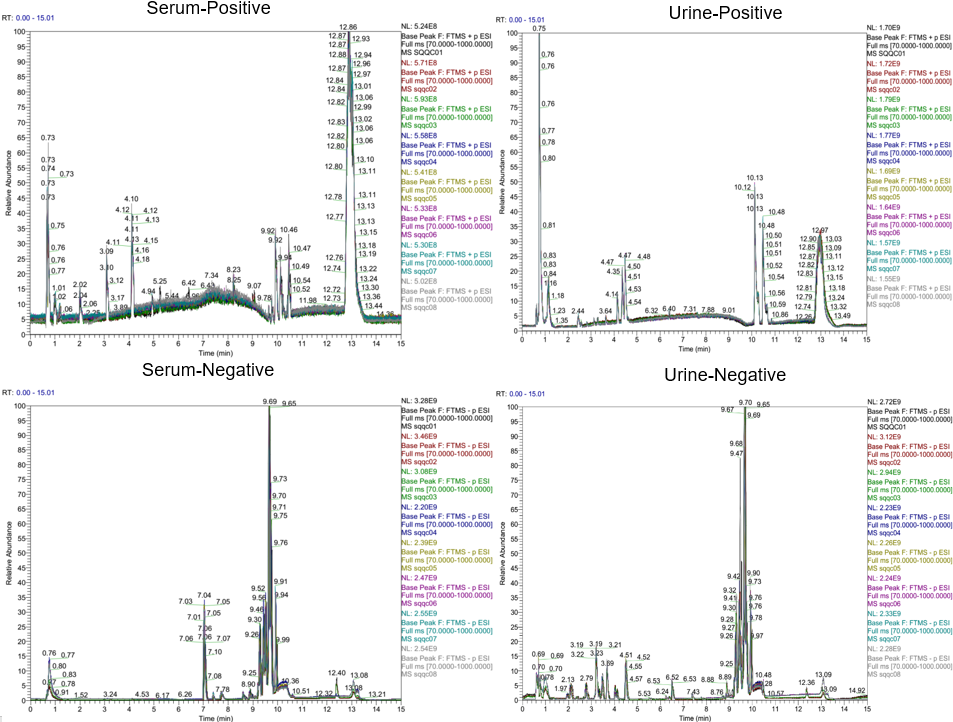


**Fig. S2.** Total ion map of serum and urine metabolomics from Biqi and LEF groups in both positive and negative ion modes as quality control assessment.


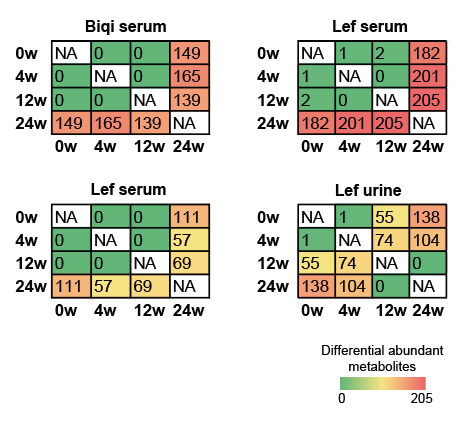


**Fig. S3.** Differentially abundant serum and urine metabolites in paired statistical comparisons in between different timepoints.


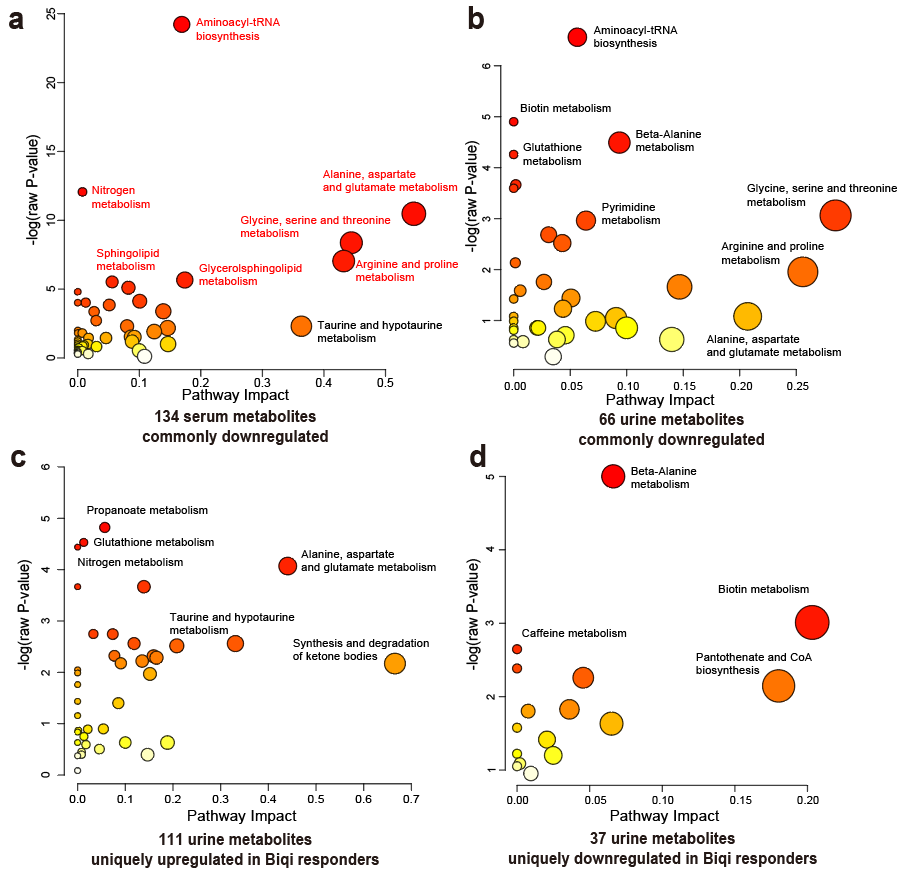


**Fig. S4.** Metabolic pathway analysis for a) 134 serum metabolites commonly downregulated at 24 weeks versus baseline in Biqi and LEF groups, b) 66 urine metabolites commonly downregulated at 24 weeks versus baseline in Biqi and LEF groups, c) 111 urine metabolites uniquely upregulated in Biqi responders versus non-responders at 24 weeks, d) 37 urine metabolites uniquely downregulated in Biqi responders versus non-responders at 24 weeks. Each dot represents one pathway colored by the raw P-value for its enrichment. The Y-axis represents the minus log (raw P-value). The X-axis represents pathway impact value from pathway topology analysis in MetaboAnalyst[5]. The size of the dot is proportional to the pathway impact value. The descriptions of the top pathways are indicated and statistical significant pathways (FDR P<0.05) are highlighted in red.


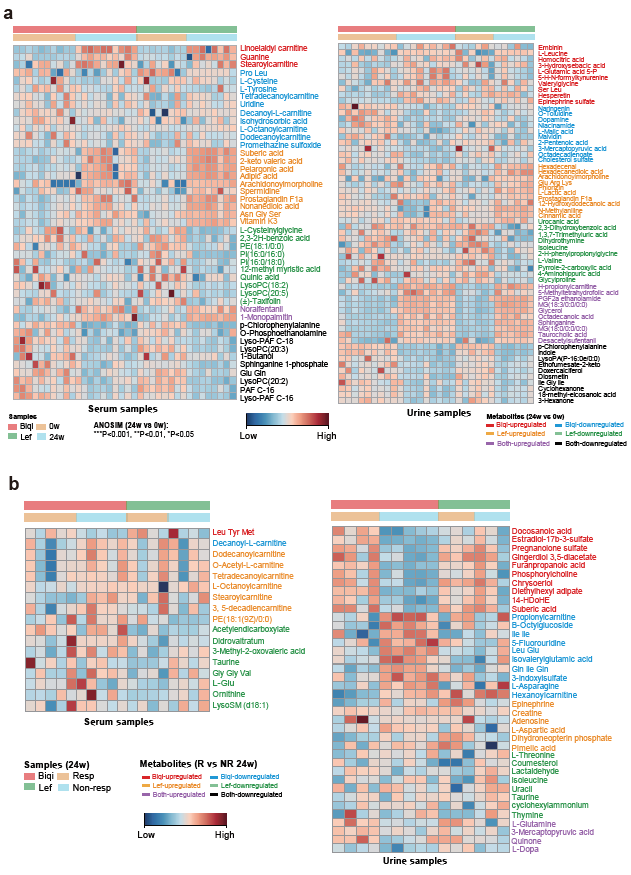


**Fig. S5.** Heatmaps for a) significantly up or downregulated serum and urine metabolites comparing 24 weeks versus baseline that were unique in Biqi or LEF group or shared in both groups, b) significantly up or downregulated serum or urine metabolites comparing responders versus non-responders at 24 weeks that were unique in Biqi or LEF group or shared in both groups. Only top 10 metabolites are shown for each comparison for visualization purpose.


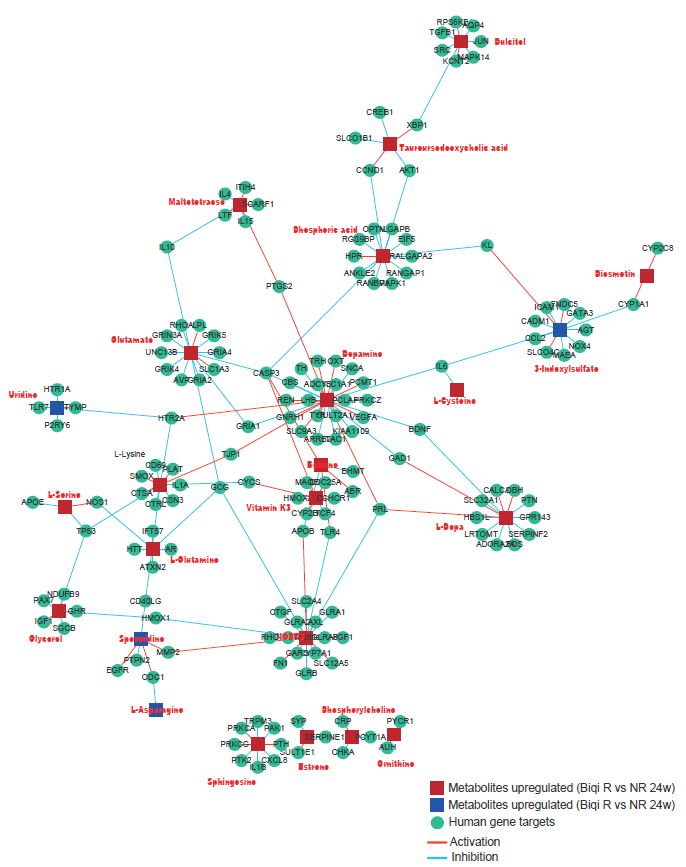


**Fig. S6.** Interaction network between differentially abundant metabolites in Biqi responders at 24 weeks and their predicted human gene targets in STITCH database[6]. Each dot in square shape represents one metabolite colored by direction of changes in Biqi responders versus non-responders at 24 weeks. Each dot in round shape represents one human gene targets. Each edge represents a metabolite-target interaction colored by activation or inhibition.


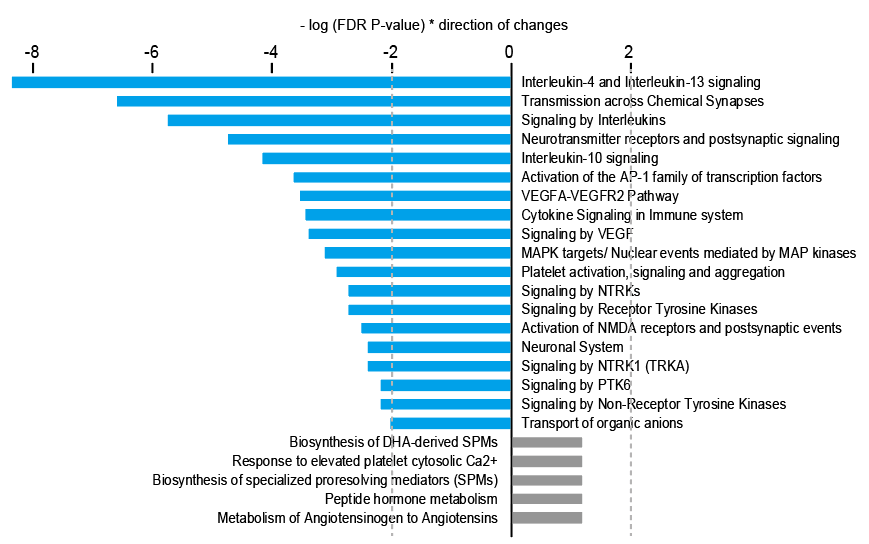


**Fig. S7.** The significantly enriched pathways for the human gene targets inferred to be upregulated or downregulated in Biqi responders versus non-responders at 24 weeks. Nineteen pathways colored in blue were significantly enriched for downregulated genes (FDR *P*<0.01). The top pathway was interleukin-4 and interleukin-13 signaling pathway. No pathway was statistical significantly enriched for upregulated genes. The top five pathways for the upregulated genes are shown (colored in grey).

**Table S1.** Patient clinical measurements of Biqi and LEF groups at each visit.

| **Characteristics** | **MTX+Biqi** | | | | **MTX+LEF** | | | |
| --- | --- | --- | --- | --- | --- | --- | --- | --- |
|  | **0w** | **4w** | **12w** | **24w** | **0w** | **4w** | **12w** | **24w** |
| TJC,n | 9.06 (6.56) | 4.97 (4.38) | 3.19 (3.55) | 2.00 (1.88) | 7.67 (6.39) | 3.74 (3.43) | 2.67 (2.63) | 1.52 (1.55) |
| SJC,n | 5.97 (5.52) | 3.13 (4.40) | 1.47 (2.46) | 0.38 (0.83) | 4.33 (3.20) | 1.89 (2.75) | 1.48 (2.33) | 0.67 (1.07) |
| Patient's assessment of pain(SD),mm† | 56.09 (24.12) | 42.19 (20.00) | 25.78 (16.02) | 21.09 (13.18) | 57.78 (20.06) | 40.19 (18.21) | 28.26 (16.70) | 20.19 (15.47) |
| Physician's assessment of disease activity(SD),mm† | 49.78 (25.21) | 41.41 (20.29) | 25.16 (15.37) | 21.09 (12.93) | 53.33 (19.17) | 37.04 (19.13) | 26.85 (16.36) | 17.41 (15.28) |
| Patient's assessment of disease activity(SD),mm† | 54.06 (25.67) | 42.34 (20.04) | 26.72 (18.12) | 22.03 (13.90) | 53.89 (19.03) | 41.30 (19.98) | 27.04 (17.50) | 17.59 (14.63) |
| Morning stiffness(min) | 67.94 (53.60) | 39.06 (42.72) | 13.45 (33.80) | 6.00 (15.66) | 54.42 (48.19) | 40.38 (48.64) | 14.73 (17.34) | 7.04 (23.43) |
| CRP, mg/L | 9.38 (10.41) | 11.30 (19.08) | 5.52 (7.19) | 5.99 (8.28) | 17.20 (18.55) | 11.37 (14.16) | 8.96 (10.51) | 7.29 (10.34) |
| ESR, mm/h | 42.66 (26.32) | 43.65 (24.99) | 35.66 (18.75) | 37.57 (24.51) | 55.00 (27.85) | 46.44 (28.51) | 41.85 (28.05) | 39.22 (29.09) |
| RF, U/mL | 146.57 (166.50) | 122.39 (133.63) | 127.32 (156.28) | 216.01 (575.06) | 159.76 (170.93) | 121.06 (146.68) | 88.43 (114.24) | 86.88 (114.30) |
| HAQ | 0.99 (0.81) | 0.58 (0.61) | 0.30 (0.52) | 0.21 (0.45) | 0.74 (0.76) | 0.40 (0.54) | 0.31 (0.38) | 0.14 (0.26) |

Values are the mean,SD. TJC, tender joint count; SJC, swollen joint count; PaGADA, patient’s global assessment of disease activity; PhGADA, physician’s global assessment of disease activity; HAQ, Health Assessment Questionnaire; ESR, erythrocyte sedimentation rate; CRP, C-reactive protein; RF, rheumatoid factor; DAS28, 28-joint disease activity score.

† Measured on a 100-mm visual analog scale.

**Table S2.** Overview of patient serum and urine metabolomic sampling in this pilot study.

| **Subject** | **PP analysis** | **Responder** | **Serum metabolomics** | | | | **Urine metabolomics** | | | |
| --- | --- | --- | --- | --- | --- | --- | --- | --- | --- | --- |
|  |  |  | **0w** | **4w** | **16w** | **24w** | **0w** | **4w** | **16w** | **24w** |
| BM001 | 1 | 1 | 1 | 1 | 1 | 1 | 1 | 1 | 1 | 1 |
| BM002 | 1 | 1 | 1 | 0 | 1 | 1 | 1 | 1 | 1 | 1 |
| BM003 | 1 | 1 | 1 | 1 | 1 | 0 | 0 | 1 | 1 | 1 |
| BM004 | 1 | 1 | 1 | 1 | 1 | 1 | 1 | 1 | 1 | 0 |
| BM005 | 0 | NA | 1 | 1 | 0 | 0 | 1 | 1 | 0 | 0 |
| BM006 | 1 | 0 | 1 | 0 | 0 | 1 | 1 | 0 | 0 | 1 |
| BM007 | 1 | 0 | 1 | 1 | 1 | 1 | 1 | 1 | 1 | 1 |
| BM008 | 1 | 1 | 1 | 0 | 1 | 0 | 1 | 0 | 1 | 1 |
| BM009 | 1 | 1 | 1 | 1 | 1 | 1 | 0 | 1 | 1 | 1 |
| BM010 | 1 | 0 | 0 | 1 | 1 | 1 | 0 | 1 | 1 | 1 |
| BM012 | 1 | 0 | 1 | 1 | 1 | 1 | 1 | 1 | 1 | 1 |
| BM013 | 1 | 1 | 1 | 1 | 1 | 1 | 1 | 1 | 1 | 1 |
| BM014 | 1 | 1 | 1 | 1 | 1 | 0 | 0 | 1 | 1 | 0 |
| BM015 | 1 | 0 | 1 | 1 | 1 | 1 | 1 | 1 | 1 | 1 |
| BM016 | 1 | 1 | 1 | 1 | 1 | 0 | 1 | 1 | 1 | 1 |
| BM017 | 1 | 1 | 1 | 1 | 1 | 0 | 1 | 1 | 1 | 0 |
| BM018 | 1 | 0 | 1 | 1 | 1 | 0 | 1 | 1 | 1 | 0 |
| BM022 | 0 | NA | 0 | 0 | 0 | 0 | 0 | 0 | 0 | 0 |
| BM023 | 0 | NA | 0 | 0 | 0 | 0 | 0 | 0 | 0 | 0 |
| BM024 | 1 | 0 | 0 | 0 | 0 | 0 | 0 | 0 | 0 | 0 |
| BM025 | 1 | 0 | 0 | 0 | 0 | 0 | 0 | 0 | 0 | 0 |
| BM026 | 1 | 1 | 0 | 0 | 0 | 0 | 0 | 0 | 0 | 0 |
| BM027 | 1 | 1 | 0 | 0 | 0 | 0 | 0 | 0 | 0 | 0 |
| BM028 | 1 | 0 | 0 | 0 | 0 | 0 | 0 | 0 | 0 | 0 |
| BM029 | 1 | 0 | 0 | 0 | 0 | 0 | 0 | 0 | 0 | 0 |
| BM030 | 1 | 1 | 0 | 0 | 0 | 0 | 0 | 0 | 0 | 0 |
| BM031 | 1 | 0 | 0 | 0 | 0 | 0 | 0 | 0 | 0 | 0 |
| BM032 | 1 | 0 | 0 | 0 | 0 | 0 | 0 | 0 | 0 | 0 |
| BM033 | 1 | 0 | 0 | 0 | 0 | 0 | 0 | 0 | 0 | 0 |
| BM034 | 1 | 0 | 0 | 0 | 0 | 0 | 0 | 0 | 0 | 0 |
| BM035 | 1 | 1 | 0 | 0 | 0 | 0 | 0 | 0 | 0 | 0 |
| BM036 | 1 | 1 | 0 | 0 | 0 | 0 | 0 | 0 | 0 | 0 |
| BM037 | 1 | 0 | 0 | 0 | 0 | 0 | 0 | 0 | 0 | 0 |
| BM038 | 1 | 0 | 0 | 0 | 0 | 0 | 0 | 0 | 0 | 0 |
| BM039 | 1 | 0 | 0 | 0 | 0 | 0 | 0 | 0 | 0 | 0 |
| LM003 | 0 | NA | 0 | 1 | 0 | 0 | 1 | 0 | 0 | 0 |
| LM006 | 1 | 1 | 1 | 1 | 1 | 1 | 1 | 1 | 1 | 1 |
| LM007 | 0 | NA | 1 | 1 | 1 | 1 | 1 | 1 | 0 | 0 |
| LM010 | 1 | 0 | 1 | 1 | 1 | 1 | 1 | 1 | 1 | 1 |
| LM013 | 1 | 0 | 1 | 1 | 1 | 1 | 1 | 1 | 1 | 1 |
| LM014 | 1 | 1 | 1 | 1 | 1 | 1 | 1 | 1 | 1 | 1 |
| LM016 | 1 | 1 | 1 | 1 | 1 | 1 | 0 | 1 | 1 | 1 |
| LM017 | 1 | 0 | 1 | 1 | 1 | 1 | 0 | 1 | 1 | 1 |
| LM018 | 1 | 1 | 1 | 1 | 1 | 0 | 1 | 1 | 1 | 1 |
| LM019 | 1 | 0 | 1 | 1 | 1 | 1 | 1 | 1 | 1 | 1 |
| LM020 | 1 | 1 | 1 | 1 | 1 | 0 | 1 | 1 | 1 | 0 |
| LM024 | 1 | 1 | 1 | 1 | 1 | 0 | 1 | 1 | 1 | 0 |
| LM025 | 1 | 1 | 1 | 1 | 1 | 0 | 1 | 1 | 1 | 0 |
| LM026 | 1 | 1 | 1 | 1 | 0 | 0 | 1 | 1 | 0 | 0 |
| LM027 | 1 | 1 | 1 | 1 | 0 | 0 | 1 | 1 | 0 | 0 |
| LM028 | 1 | 0 | 1 | 1 | 0 | 0 | 1 | 1 | 0 | 0 |
| LM030 | 0 | NA | 0 | 0 | 0 | 0 | 0 | 0 | 0 | 0 |
| LM031 | 0 | NA | 0 | 0 | 0 | 0 | 0 | 0 | 0 | 0 |
| LM032 | 0 | NA | 0 | 0 | 0 | 0 | 0 | 0 | 0 | 0 |
| LM033 | 0 | NA | 0 | 0 | 0 | 0 | 0 | 0 | 0 | 0 |
| LM034 | 0 | NA | 0 | 0 | 0 | 0 | 0 | 0 | 0 | 0 |
| LM035 | 1 | 0 | 0 | 0 | 0 | 0 | 0 | 0 | 0 | 0 |
| LM036 | 1 | 1 | 0 | 0 | 0 | 0 | 0 | 0 | 0 | 0 |
| LM037 | 1 | 0 | 0 | 0 | 0 | 0 | 0 | 0 | 0 | 0 |
| LM038 | 1 | 1 | 0 | 0 | 0 | 0 | 0 | 0 | 0 | 0 |
| LM039 | 1 | 0 | 0 | 0 | 0 | 0 | 0 | 0 | 0 | 0 |
| LM040 | 1 | 1 | 0 | 0 | 0 | 0 | 0 | 0 | 0 | 0 |
| LM041 | 1 | 0 | 0 | 0 | 0 | 0 | 0 | 0 | 0 | 0 |
| LM042 | 1 | 1 | 0 | 0 | 0 | 0 | 0 | 0 | 0 | 0 |
| LM043 | 1 | 1 | 0 | 0 | 0 | 0 | 0 | 0 | 0 | 0 |
| LM044 | 1 | 0 | 0 | 0 | 0 | 0 | 0 | 0 | 0 | 0 |
| LM045 | 1 | 0 | 0 | 0 | 0 | 0 | 0 | 0 | 0 | 0 |
| LM046 | 1 | 0 | 0 | 0 | 0 | 0 | 0 | 0 | 0 | 0 |
| LM047 | 1 | 0 | 0 | 0 | 0 | 0 | 0 | 0 | 0 | 0 |
| LM048 | 0 | NA | 0 | 0 | 0 | 0 | 0 | 0 | 0 | 0 |

**Table S3.** 134 serum and 66 urine metabolites that were significantly downregulated in common between Biqi and LEF groups, comparing 24 weeks versus baseline.

| **Metabolite** | **Biqi FC** | **Biqi FDR** | **LEF FC** | **LEF FDR** | **Serum /Urine** |
| --- | --- | --- | --- | --- | --- |
| P-Chlorophenylalanine | -19.26 | 7.19E-06 | -17.52 | 3.78E-05 | Serum |
| O-Phosphoethanolamine | -15.76 | 1.74E-03 | -10.33 | 7.23E-03 | Serum |
| Lyso-PAF C-18 | -14.55 | 3.29E-03 | -11.78 | 3.42E-04 | Serum |
| LysoPC(20:3(5Z,8Z,11Z)) | -13.00 | 1.04E-03 | -15.49 | 6.02E-05 | Serum |
| 1-Butanol | -12.82 | 2.55E-02 | -9.08 | 1.55E-02 | Serum |
| Sphinganine 1-phosphate | -12.68 | 1.37E-02 | -12.55 | 2.85E-04 | Serum |
| Glu Gln | -12.44 | 1.04E-03 | -12.75 | 2.12E-04 | Serum |
| LysoPC(20:2(11Z,14Z)) | -12.24 | 3.60E-03 | -17.72 | 6.02E-05 | Serum |
| PAF C-16 | -12.17 | 1.04E-03 | -15.23 | 8.70E-05 | Serum |
| Lyso-PAF C-16 | -12.10 | 3.29E-03 | -13.39 | 1.13E-04 | Serum |
| Choline | -12.08 | 3.24E-03 | -16.99 | 3.78E-05 | Serum |
| 1-Oleoyl Lysophosphatidic Acid | -12.01 | 2.06E-03 | -14.81 | 4.11E-05 | Serum |
| L-threonic Acid | -11.85 | 7.92E-03 | -12.22 | 2.92E-04 | Serum |
| LysoPC(17:0) | -11.75 | 1.65E-03 | -14.72 | 1.95E-04 | Serum |
| 1-Palmitoyl Lysophosphatidic Acid | -11.72 | 1.66E-03 | -14.87 | 3.78E-05 | Serum |
| LysoPC(16:0) | -11.65 | 2.15E-03 | -15.12 | 2.44E-04 | Serum |
| L-Methionine | -11.64 | 1.66E-03 | -10.73 | 9.92E-04 | Serum |
| Sphingosine 1-phosphate | -11.63 | 1.14E-02 | -11.81 | 1.02E-03 | Serum |
| L-Threonine | -11.57 | 1.87E-03 | -11.20 | 5.75E-04 | Serum |
| L-arginine | -11.47 | 1.04E-03 | -10.35 | 6.13E-04 | Serum |
| Cysteine-S-sulfate | -11.44 | 5.60E-03 | -10.95 | 8.65E-04 | Serum |
| Dihydroxyacetone | -11.39 | 1.04E-03 | -11.83 | 2.01E-02 | Serum |
| 2-Methylbutyroylcarnitine | -11.39 | 1.84E-02 | -11.64 | 3.16E-04 | Serum |
| Glycolic acid | -11.37 | 1.19E-02 | -6.07 | 3.92E-02 | Serum |
| L-Lysine | -11.29 | 1.66E-03 | -10.74 | 3.61E-04 | Serum |
| LysoPA(P-16:0e/0:0) | -11.14 | 1.57E-02 | -12.10 | 5.57E-04 | Serum |
| Cycloleucine | -11.06 | 5.09E-03 | -9.61 | 9.01E-04 | Serum |
| LysoPC(15:0) | -10.94 | 1.66E-03 | -14.09 | 3.78E-05 | Serum |
| LysoPC(P-18:0) | -10.83 | 7.91E-03 | -12.71 | 1.95E-04 | Serum |
| Glycolic acid | -10.78 | 2.06E-03 | -13.22 | 3.84E-03 | Serum |
| Acetylglycine | -10.77 | 1.19E-02 | -10.70 | 7.23E-03 | Serum |
| Methionine | -10.77 | 1.74E-03 | -7.04 | 1.99E-02 | Serum |
| Glutarylcarnitine | -10.73 | 7.97E-03 | -8.05 | 6.13E-04 | Serum |
| LysoPC(16:0) | -10.72 | 1.66E-03 | -13.87 | 8.85E-05 | Serum |
| 1-Methylhistidine | -10.71 | 2.06E-03 | -8.85 | 5.41E-03 | Serum |
| Creatine | -10.67 | 1.50E-03 | -8.71 | 4.06E-03 | Serum |
| LysoPC(18:1(9Z)) | -10.62 | 2.55E-03 | -14.78 | 1.13E-04 | Serum |
| cholesterol sulfate | -10.56 | 1.12E-03 | -13.62 | 3.78E-05 | Serum |
| 1-Methylhistidine | -10.53 | 1.65E-03 | -10.60 | 2.45E-04 | Serum |
| Hydrosorbic acid | -10.53 | 3.60E-03 | -12.02 | 2.90E-03 | Serum |
| Glycine | -10.44 | 3.60E-03 | -11.75 | 8.65E-04 | Serum |
| LysoPC(16:1(9Z)/0:0) | -10.38 | 2.55E-03 | -12.06 | 1.95E-04 | Serum |
| LysoPC(20:0/0:0) | -10.35 | 2.06E-03 | -15.54 | 1.88E-03 | Serum |
| Glycine | -10.33 | 1.82E-03 | -13.86 | 3.78E-05 | Serum |
| Decanoylcarnitine | -10.32 | 3.33E-03 | -11.94 | 6.02E-05 | Serum |
| Tyrosine | -10.29 | 1.66E-03 | -11.14 | 2.91E-04 | Serum |
| L-Asparagine | -10.25 | 2.55E-03 | -12.06 | 1.62E-04 | Serum |
| L-Threonine | -10.16 | 3.29E-03 | -9.19 | 2.18E-03 | Serum |
| Glycerol tributanoate | -10.15 | 6.92E-03 | -10.44 | 1.56E-02 | Serum |
| Xanthine | -10.13 | 1.84E-02 | -8.84 | 6.63E-04 | Serum |
| LysoPC(17:0) | -10.11 | 1.74E-03 | -15.55 | 1.95E-04 | Serum |
| Cer(d18:1/14:0) | -9.94 | 1.15E-02 | -11.06 | 3.37E-03 | Serum |
| L-Glu | -9.90 | 1.06E-02 | -12.11 | 2.19E-04 | Serum |
| Uracil | -9.89 | 5.37E-03 | -13.68 | 3.78E-05 | Serum |
| L-Serine | -9.86 | 4.55E-03 | -10.73 | 6.88E-04 | Serum |
| Glycerol 3-phosphate | -9.73 | 1.41E-02 | -12.36 | 3.68E-04 | Serum |
| Leu His Arg | -9.73 | 8.72E-03 | -7.54 | 1.63E-02 | Serum |
| Benzoic acid | -9.67 | 1.71E-03 | -11.68 | 2.06E-04 | Serum |
| Diosmetin | -9.64 | 7.09E-03 | -7.35 | 1.07E-02 | Serum |
| L-Alanine | -9.63 | 3.60E-03 | -11.60 | 2.91E-04 | Serum |
| LysoPC(18:0) | -9.55 | 6.05E-03 | -16.91 | 3.37E-03 | Serum |
| L-Histidine | -9.53 | 6.08E-03 | -11.93 | 2.90E-03 | Serum |
| Glutamate | -9.39 | 1.47E-02 | -11.95 | 2.19E-04 | Serum |
| Acetylglycine | -9.30 | 7.92E-03 | -6.95 | 1.30E-03 | Serum |
| LysoPC(22:6(4Z,7Z,10Z,13Z,16Z,19Z)) | -9.29 | 1.65E-03 | -12.84 | 2.87E-04 | Serum |
| L-Valine | -9.20 | 2.15E-03 | -9.24 | 7.38E-04 | Serum |
| L-Pyroglutamic acid | -9.14 | 5.60E-03 | -12.87 | 1.95E-04 | Serum |
| Ornithine | -9.06 | 3.08E-03 | -11.22 | 4.11E-05 | Serum |
| 6-Thioxanthine | -8.95 | 2.45E-02 | -8.27 | 1.09E-03 | Serum |
| MG(0:0/18:2(9Z,12Z)/0:0) | -8.93 | 1.95E-02 | -8.36 | 2.79E-03 | Serum |
| Betaine | -8.92 | 3.97E-03 | -9.38 | 1.85E-03 | Serum |
| Malic acid | -8.88 | 2.65E-02 | -12.15 | 6.02E-05 | Serum |
| 2-Hepten-4-yn-1-amine | -8.83 | 6.92E-03 | -10.04 | 5.57E-04 | Serum |
| LysoPC(22:5(7Z,10Z,13Z,16Z,19Z)) | -8.81 | 6.38E-03 | -10.47 | 1.56E-02 | Serum |
| Xanthine | -8.79 | 2.32E-02 | -8.77 | 7.67E-04 | Serum |
| Citrate | -8.75 | 3.09E-02 | -11.07 | 4.55E-04 | Serum |
| Propynoic acid | -8.74 | 1.66E-03 | -14.23 | 1.79E-04 | Serum |
| Granisetron | -8.71 | 5.09E-03 | -9.29 | 2.23E-03 | Serum |
| His | -8.64 | 7.02E-03 | -11.13 | 2.64E-04 | Serum |
| Taurine | -8.63 | 2.59E-03 | -10.77 | 6.02E-05 | Serum |
| L-Proline | -8.61 | 6.81E-03 | -10.55 | 2.85E-04 | Serum |
| Phosphorylcholine | -8.44 | 1.54E-02 | -11.38 | 5.28E-04 | Serum |
| PGF2alpha-d4 | -8.32 | 2.44E-03 | -10.24 | 5.68E-03 | Serum |
| O-Acetyl-L-carnitine | -8.22 | 5.47E-03 | -5.03 | 1.98E-02 | Serum |
| 5'-Methylthioadenosine | -8.21 | 6.92E-03 | -10.21 | 4.76E-03 | Serum |
| Tryptophan | -8.19 | 7.15E-03 | -10.54 | 8.85E-05 | Serum |
| Norethindrone acetate | -8.17 | 2.07E-03 | -10.64 | 3.78E-05 | Serum |
| Guanidinosuccinic acid | -8.13 | 2.22E-02 | -5.90 | 8.65E-04 | Serum |
| L-Glutamine | -8.10 | 3.29E-03 | -10.06 | 5.28E-04 | Serum |
| Tryptophan | -8.09 | 4.40E-03 | -10.19 | 1.80E-04 | Serum |
| L-Arabitol | -8.03 | 5.69E-03 | -10.23 | 3.78E-05 | Serum |
| Gamma-Glu-Leu | -8.01 | 5.09E-03 | -9.89 | 8.13E-04 | Serum |
| Creatinine | -7.97 | 1.04E-02 | -11.05 | 1.14E-04 | Serum |
| PC(14:0/0:0) | -7.96 | 6.92E-03 | -8.79 | 1.23E-04 | Serum |
| Uric acid | -7.95 | 4.10E-02 | -7.41 | 1.90E-03 | Serum |
| Ornithine | -7.86 | 3.29E-03 | -9.32 | 2.91E-04 | Serum |
| Dopamine | -7.72 | 3.33E-03 | -11.11 | 3.78E-05 | Serum |
| Lansoprazole | -7.71 | 1.89E-03 | -9.36 | 3.78E-05 | Serum |
| LysoPC(18:3(6Z,9Z,12Z)) | -7.68 | 1.74E-03 | -11.57 | 5.57E-04 | Serum |
| L-Lysine | -7.65 | 5.12E-03 | -9.41 | 6.49E-04 | Serum |
| L-Valine | -7.56 | 7.15E-03 | -10.49 | 3.09E-04 | Serum |
| 1,2-Dihydrosantonin | -7.54 | 1.66E-03 | -8.83 | 7.90E-04 | Serum |
| L-Histidine | -7.52 | 1.99E-03 | -9.06 | 3.68E-04 | Serum |
| D-Carnitine | -7.50 | 7.92E-03 | -9.16 | 1.34E-03 | Serum |
| L-Serine | -7.41 | 4.12E-02 | -9.19 | 7.47E-03 | Serum |
| Isoleucine | -7.29 | 1.41E-02 | -12.44 | 2.19E-04 | Serum |
| Dulcitol | -7.24 | 1.23E-02 | -9.79 | 8.85E-05 | Serum |
| Eicosadienoic acid | -7.23 | 7.60E-03 | -7.83 | 6.63E-04 | Serum |
| Fumaric acid | -7.13 | 3.85E-02 | -11.49 | 6.02E-05 | Serum |
| L-(-)-Phenylalanine | -7.11 | 1.29E-02 | -10.59 | 3.09E-04 | Serum |
| 4' 5 7-trihydroxyflavanone | -7.03 | 1.66E-03 | -7.47 | 1.38E-04 | Serum |
| Propionylcarnitine | -6.89 | 2.19E-02 | -8.04 | 2.08E-03 | Serum |
| Glycerol tributanoate | -6.86 | 3.60E-03 | -5.92 | 2.24E-02 | Serum |
| Resveratrol | -6.76 | 1.89E-03 | -6.79 | 2.19E-04 | Serum |
| Androsterone glucuronide | -6.69 | 1.22E-02 | -8.37 | 1.14E-02 | Serum |
| L-Proline | -6.66 | 1.95E-02 | -8.55 | 1.02E-03 | Serum |
| Glucose | -6.47 | 2.07E-02 | -8.54 | 1.02E-03 | Serum |
| 3-Chlorotyrosine | -6.47 | 2.28E-02 | -7.90 | 4.13E-02 | Serum |
| PC(P-16:0/18:4(6Z,9Z,12Z,15Z)) | -6.42 | 2.14E-02 | -11.66 | 8.65E-04 | Serum |
| Docosahexaenoic acid | -6.40 | 3.45E-02 | -5.02 | 7.30E-03 | Serum |
| b-Hydroxypropionate | -6.30 | 2.32E-02 | -8.68 | 6.29E-05 | Serum |
| Succinic acid semialdehyde | -6.06 | 1.77E-02 | -7.55 | 3.78E-05 | Serum |
| Ornithine | -6.03 | 2.22E-02 | -10.46 | 1.45E-04 | Serum |
| Gluconic acid | -5.83 | 2.09E-02 | -16.66 | 1.30E-03 | Serum |
| Uridine | -5.80 | 6.81E-03 | -8.92 | 1.79E-04 | Serum |
| L-Arabinose | -5.69 | 3.49E-02 | -9.34 | 7.16E-04 | Serum |
| (±)9-HODE | -5.47 | 2.20E-02 | -7.66 | 8.85E-05 | Serum |
| Succinyladenosine | -5.43 | 4.53E-02 | -5.78 | 1.75E-02 | Serum |
| (+)-18-methyl-eicosanoic acid | -5.35 | 2.51E-02 | -10.02 | 2.12E-04 | Serum |
| 2-furanpropanoic acid | -5.19 | 1.26E-02 | -6.60 | 1.50E-04 | Serum |
| PGD2 ethanolamide | -4.78 | 2.57E-02 | -2.29 | 3.13E-02 | Serum |
| m-chlorophenylpiperazine | -4.25 | 3.09E-02 | -6.65 | 3.55E-02 | Serum |
| Tricosanoic acid | -4.20 | 3.09E-02 | -5.23 | 3.76E-02 | Serum |
| Sphingosine-1-phosphate | -3.38 | 2.65E-02 | -5.85 | 1.79E-04 | Serum |
| P-Chlorophenylalanine | -19.61 | 5.75E-07 | -11.69 | 4.94E-07 | Urine |
| Indole | -19.47 | 7.51E-06 | -10.58 | 3.39E-05 | Urine |
| LysoPA(P-16:0e/0:0) | -15.52 | 2.56E-04 | -5.84 | 3.99E-04 | Urine |
| Ethofumesate-2-keto | -15.44 | 7.92E-03 | -3.84 | 2.49E-02 | Urine |
| Doxercalciferol | -13.93 | 6.61E-03 | -6.57 | 8.95E-04 | Urine |
| Diosmetin | -13.72 | 7.73E-03 | -5.57 | 3.86E-02 | Urine |
| Ile Gly Ile | -13.42 | 3.46E-03 | -5.89 | 8.96E-03 | Urine |
| Cyclohexanone | -12.91 | 3.19E-03 | -3.84 | 2.91E-02 | Urine |
| Eicosanoic acid | -12.81 | 5.22E-03 | -3.79 | 4.33E-02 | Urine |
| 3-Hexanone | -12.65 | 6.63E-04 | -5.26 | 4.07E-02 | Urine |
| Norethindrone acetate | -12.60 | 4.39E-03 | -4.10 | 4.22E-02 | Urine |
| Cloransulam-methyl | -12.53 | 5.46E-03 | -4.94 | 1.55E-02 | Urine |
| Ribitol | -12.18 | 6.18E-03 | -9.33 | 1.36E-03 | Urine |
| Acaciin | -12.05 | 1.66E-02 | -4.16 | 4.20E-02 | Urine |
| N,N'-diacetylchitobiose | -11.90 | 3.59E-02 | -5.84 | 3.33E-02 | Urine |
| Desogestrel | -11.81 | 5.41E-03 | -3.73 | 3.84E-02 | Urine |
| Estradiol-17beta 3-sulfate | -11.71 | 4.98E-03 | -2.88 | 3.06E-02 | Urine |
| Propynoic acid | -11.37 | 3.14E-03 | -6.49 | 8.27E-03 | Urine |
| Isobutyric acid | -10.65 | 3.52E-03 | -5.24 | 3.30E-02 | Urine |
| Allocystathionine | -10.42 | 1.23E-02 | -4.26 | 4.03E-02 | Urine |
| Cytosine | -10.04 | 1.77E-02 | -7.35 | 9.48E-03 | Urine |
| Adenine | -9.99 | 1.13E-04 | -2.86 | 3.95E-02 | Urine |
| 15-methyl palmitic acid | -8.08 | 2.43E-03 | -5.04 | 3.06E-02 | Urine |
| Butyrylcarnitine | -7.76 | 4.02E-03 | -8.58 | 4.41E-04 | Urine |
| Docosahexaenoic acid | -7.46 | 1.66E-03 | -5.34 | 1.02E-02 | Urine |
| Glutamine | -7.45 | 5.40E-03 | -8.45 | 5.00E-03 | Urine |
| Methylguanidine | -7.27 | 9.84E-05 | -8.02 | 1.28E-02 | Urine |
| Tauroursodeoxycholic acid | -7.03 | 2.35E-02 | -5.05 | 1.24E-02 | Urine |
| L-Pyroglutamic acid | -6.88 | 1.09E-02 | -7.57 | 8.26E-03 | Urine |
| L-Octanoylcarnitine | -6.80 | 1.53E-02 | -4.83 | 9.67E-03 | Urine |
| Pimelic acid | -6.64 | 2.73E-02 | -7.91 | 1.09E-02 | Urine |
| Gamma-Butyrolactone | -6.56 | 2.88E-02 | -1.32 | 3.76E-02 | Urine |
| b-Hydroxypropionate | -6.54 | 4.55E-02 | -2.54 | 3.98E-02 | Urine |
| Thiodiacetic acid | -6.53 | 2.56E-02 | -6.58 | 6.64E-03 | Urine |
| DL-a-Hydroxybutyric acid | -6.45 | 4.84E-02 | -2.48 | 2.41E-02 | Urine |
| 4-Methylpentanal | -6.44 | 3.12E-02 | -5.68 | 1.24E-02 | Urine |
| 5'-Hydroxylornoxicam | -6.37 | 4.68E-02 | -6.44 | 3.39E-03 | Urine |
| Docosanoic acid | -6.37 | 1.61E-02 | -5.83 | 3.17E-03 | Urine |
| Estrone | -6.28 | 2.63E-02 | -2.62 | 3.91E-02 | Urine |
| Decanoic acid | -6.26 | 1.56E-02 | -4.38 | 1.98E-02 | Urine |
| d[-Arg-2]Kyotorphan | -6.15 | 3.63E-02 | 2.96 | 3.80E-04 | Urine |
| 2-hydroxyhexadecanoic acid | -6.12 | 1.51E-02 | -4.37 | 1.06E-02 | Urine |
| Glycolic acid | -5.93 | 4.64E-02 | -9.67 | 3.72E-03 | Urine |
| Pentanal | -5.76 | 1.79E-02 | -3.98 | 2.79E-02 | Urine |
| Metanephrine | -5.75 | 3.84E-02 | -6.41 | 1.40E-02 | Urine |
| Ornithine | -5.73 | 4.44E-02 | -7.83 | 8.11E-03 | Urine |
| Arginine | -5.64 | 4.78E-02 | -8.15 | 8.95E-03 | Urine |
| L-Threonine | -5.60 | 4.98E-02 | -7.61 | 2.59E-03 | Urine |
| Glycine | -5.56 | 3.98E-02 | -4.31 | 3.11E-02 | Urine |
| Cycloleucine | -5.50 | 8.69E-03 | -11.09 | 4.87E-03 | Urine |
| Phosphorylcholine | -5.43 | 4.26E-02 | -5.69 | 1.84E-02 | Urine |
| Chrysoeriol | -5.24 | 4.82E-02 | 3.03 | 3.45E-03 | Urine |
| L-Kynurenine | -5.21 | 2.17E-03 | -6.79 | 8.06E-03 | Urine |
| Propanecarboxylic acid | -5.03 | 4.37E-02 | -7.13 | 9.19E-03 | Urine |
| Cysteine-S-sulfate | -4.98 | 1.84E-02 | -9.37 | 1.11E-02 | Urine |
| 12,13-EODE | -4.96 | 4.40E-02 | -3.49 | 3.86E-02 | Urine |
| Methyl propenyl ketone | -4.92 | 1.72E-02 | -6.41 | 2.32E-02 | Urine |
| 2-furanpropanoic acid | -4.85 | 4.76E-02 | -3.06 | 4.85E-02 | Urine |
| L-Histidine | -4.64 | 9.97E-03 | -8.11 | 6.38E-03 | Urine |
| CE(16:0) | -4.50 | 4.74E-02 | -2.09 | 3.37E-02 | Urine |
| L-Lysine | -3.92 | 3.30E-02 | -10.88 | 4.67E-03 | Urine |
| Alanyl-Proline | -3.66 | 2.42E-02 | -8.75 | 1.10E-02 | Urine |
| L-Lysine | -3.47 | 1.38E-02 | -12.31 | 4.50E-03 | Urine |
| 2-Methylpyridine | -3.25 | 5.75E-03 | -7.80 | 1.34E-02 | Urine |
| Glyceric acid | -3.20 | 4.69E-02 | -6.30 | 2.44E-02 | Urine |
| Ethanolamine Oleate | -3.22 | 5.50E-03 | -14.00 | 7.42E-03 | Urine |

**Table S4.** 111 and 37 urine metabolites that were uniquely up or downregulated in Biqi responders versus non-responders at 24 weeks in the ANCOVA analysis.

| **Metabolite** | **FC** | **FDR** | **Direction** |
| --- | --- | --- | --- |
| Docosanoic acid | 12.30 | 2.40E-03 | Up |
| Estradiol-17beta 3-sulfate | 12.13 | 4.71E-04 | Up |
| Pregnanolone sulfate | 12.06 | 6.80E-03 | Up |
| Gingerdiol 3,5-diacetate | 12.04 | 2.68E-02 | Up |
| 2-furanpropanoic acid | 11.97 | 1.52E-03 | Up |
| Phosphorylcholine | 11.93 | 1.48E-02 | Up |
| Chrysoeriol | 11.90 | 1.10E-02 | Up |
| Diethylhexyl adipate | 11.88 | 1.78E-03 | Up |
| Suberic acid | 11.78 | 1.21E-02 | Up |
| Decanoic acid | 11.74 | 3.83E-02 | Up |
| Trandolapril lactam | 11.42 | 1.25E-03 | Up |
| Adipic acid | 11.41 | 1.73E-03 | Up |
| Docosatrienoic acid | 11.35 | 1.23E-02 | Up |
| PGF2alpha-d4 | 11.33 | 6.71E-03 | Up |
| 2-hydroxyhexadecanoic acid | 11.29 | 3.08E-03 | Up |
| 2-Linoleoylglycerol | 11.01 | 5.62E-03 | Up |
| Nonanedioic acid | 10.90 | 1.09E-02 | Up |
| Phosphoguanidinoacetate | 10.90 | 9.08E-03 | Up |
| Eicosanoic acid | 10.80 | 4.39E-03 | Up |
| Ile Gly Ile | 10.76 | 2.54E-03 | Up |
| Dimethyl trisulfide | 10.76 | 1.06E-03 | Up |
| CE(16:0) | 10.76 | 1.38E-02 | Up |
| 9E,11E-octadecadienoate | 10.73 | 1.02E-02 | Up |
| CE(14:0) | 10.71 | 1.62E-02 | Up |
| Estrone | 10.69 | 2.59E-02 | Up |
| Palmitic amide | 10.54 | 1.39E-03 | Up |
| (+)-18-methyl-eicosanoic acid | 10.48 | 2.08E-02 | Up |
| 14-HDoHE | 10.43 | 8.08E-03 | Up |
| Thiodiacetic acid | 10.29 | 7.73E-03 | Up |
| o-Toluidine | 10.27 | 5.80E-03 | Up |
| Methyl butyrate | 10.07 | 1.31E-02 | Up |
| Varanic acid | 10.04 | 1.46E-03 | Up |
| Cyclohexanone | 9.87 | 1.91E-02 | Up |
| 2-Pentenoic acid | 9.83 | 1.14E-02 | Up |
| Norethindrone acetate | 9.81 | 1.93E-02 | Up |
| Betaine | 9.81 | 3.66E-03 | Up |
| 12,13-EODE | 9.60 | 1.95E-02 | Up |
| Succinic acid semialdehyde | 9.36 | 3.36E-02 | Up |
| Acetylendicarboxylate | 9.31 | 4.24E-03 | Up |
| Propynoic acid | 9.31 | 1.24E-02 | Up |
| Hexadecanedioic acid | 9.26 | 6.24E-03 | Up |
| Docosahexaenoic acid | 8.95 | 1.23E-02 | Up |
| 3-Hexanone | 8.95 | 2.07E-03 | Up |
| 3',4'-dihydroxyflurbiprofen | 8.67 | 8.46E-04 | Up |
| Isobutyric acid | 8.66 | 1.34E-02 | Up |
| Hexadecenal | 8.56 | 9.55E-03 | Up |
| Phosphoric acid | 8.54 | 1.33E-02 | Up |
| Cloransulam-methyl | 8.53 | 2.94E-02 | Up |
| 3-(Methylthio)-1-propanol | 8.48 | 1.78E-03 | Up |
| L-Cysteine | 8.37 | 2.87E-02 | Up |
| Glu Arg Lys | 8.27 | 7.86E-03 | Up |
| Phe Phe Lys | 8.21 | 8.67E-03 | Up |
| Sphingosine | 7.98 | 1.95E-02 | Up |
| Desogestrel | 7.97 | 2.90E-02 | Up |
| Biopterin | 7.96 | 6.84E-05 | Up |
| DL-a-Hydroxybutyric acid | 7.96 | 8.60E-03 | Up |
| Diosmetin | 7.92 | 4.82E-02 | Up |
| Doxercalciferol | 7.74 | 8.60E-03 | Up |
| Tauroursodeoxycholic acid | 7.72 | 1.94E-02 | Up |
| L-Valine | 7.71 | 6.60E-03 | Up |
| Taurine | 7.70 | 2.54E-02 | Up |
| Oxalic acid | 7.67 | 2.93E-02 | Up |
| Dopamine | 7.65 | 6.97E-03 | Up |
| Metanephrine | 7.60 | 1.72E-02 | Up |
| L-Cystine | 7.34 | 4.05E-02 | Up |
| Pimelic acid | 7.32 | 8.90E-03 | Up |
| Glucosamine 6-sulfate | 7.26 | 3.39E-02 | Up |
| Allylestrenol | 7.15 | 2.09E-02 | Up |
| cis-Aconitic acid | 7.12 | 2.59E-02 | Up |
| L-Pyroglutamic acid | 7.10 | 6.61E-03 | Up |
| LysoPA(P-16:0e/0:0) | 7.07 | 4.45E-02 | Up |
| L-Cystine | 7.01 | 2.44E-02 | Up |
| Lys Asp Tyr | 6.87 | 1.05E-02 | Up |
| 1-Oleoyl Lysophosphatidic Acid | 6.78 | 3.97E-02 | Up |
| Desacetylsufentanil | 6.57 | 3.48E-02 | Up |
| Imidazolone | 5.93 | 2.73E-02 | Up |
| Acetoacetic acid | 5.91 | 4.34E-02 | Up |
| Glutamine | 5.90 | 1.86E-02 | Up |
| Noralfentanil, Norsufentanil | 5.85 | 1.25E-02 | Up |
| L-Glutamine | 5.83 | 1.24E-02 | Up |
| 1-Aminocyclopropanecarboxylic acid | 5.60 | 1.39E-02 | Up |
| 4-Hydroxymethylimidazole | 5.57 | 4.85E-02 | Up |
| L-Serine | 5.54 | 3.72E-02 | Up |
| Taurine | 5.52 | 2.82E-02 | Up |
| Glutamate | 5.52 | 3.76E-02 | Up |
| L-Histidine | 5.47 | 1.52E-03 | Up |
| L-Lysine | 5.38 | 1.73E-02 | Up |
| 2-Methylpyridine | 5.36 | 6.76E-03 | Up |
| His Gly | 5.25 | 1.89E-02 | Up |
| Alanyl-Proline | 5.21 | 3.59E-02 | Up |
| 21-hydroxypregnenolone | 5.13 | 8.54E-03 | Up |
| P-Chlorophenylalanine | 4.96 | 3.53E-02 | Up |
| Cytosine | 4.68 | 3.53E-02 | Up |
| Malic acid | 4.68 | 7.02E-03 | Up |
| Phlorizin | 4.62 | 4.45E-02 | Up |
| Vitamin K3 | 4.51 | 3.64E-03 | Up |
| Ethanolamine Oleate | 4.47 | 8.52E-04 | Up |
| Citrate | 4.44 | 4.36E-02 | Up |
| 15-methyl palmitic acid | 4.25 | 4.56E-02 | Up |
| Dulcitol | 4.20 | 1.61E-02 | Up |
| Ornithine | 4.15 | 4.49E-02 | Up |
| Levulinic acid | 4.12 | 2.81E-02 | Up |
| Cycloleucine | 4.11 | 4.64E-02 | Up |
| Glycerol | 3.91 | 2.47E-03 | Up |
| 1-Hydroxypyrene glucuronide | 3.91 | 1.85E-02 | Up |
| Maltotetraose | 3.61 | 1.52E-03 | Up |
| Valine | 3.56 | 2.30E-02 | Up |
| MG(18:3(9Z,12Z,15Z)/0:0/0:0) | 3.21 | 2.29E-03 | Up |
| DG(18:3(9Z,12Z,15Z)/20:0/0:0)[iso2] | 2.58 | 2.72E-03 | Up |
| Urocanic acid | 2.40 | 4.01E-02 | Up |
| 6,16-dimethyl-octadecanoic acid | 1.46 | 8.34E-03 | Up |
| Lactosamine | -2.81 | 4.85E-02 | Down |
| Ile Gln | -3.61 | 4.85E-02 | Down |
| 3-Methoxytyrosine | -4.02 | 2.41E-02 | Down |
| 4-Aminohippuric acid | -4.24 | 7.26E-03 | Down |
| cis-5-Decenedioic acid | -4.27 | 3.38E-02 | Down |
| Dopamine glucuronide | -4.45 | 1.83E-02 | Down |
| N-Acryloylglycine | -4.56 | 1.68E-02 | Down |
| Porphobilinogen | -4.68 | 2.31E-02 | Down |
| Dopamine 4-sulfate | -4.92 | 3.21E-02 | Down |
| 1,3,7-Trimethyluric acid | -5.14 | 6.48E-03 | Down |
| Xanthosine | -5.28 | 4.54E-03 | Down |
| Heptanoylcarnitine | -5.29 | 1.66E-02 | Down |
| (S)-3,4-Dihydroxybutyric acid | -5.35 | 2.15E-02 | Down |
| Porphobilinogen | -5.39 | 1.23E-02 | Down |
| N-Acetylcystathionine | -5.53 | 4.53E-02 | Down |
| Pimelylcarnitine | -5.56 | 3.15E-02 | Down |
| N4-Acetylcytidine | -5.60 | 3.28E-02 | Down |
| Butenylcarnitine | -5.76 | 8.05E-04 | Down |
| Succinyladenosine | -6.00 | 1.16E-02 | Down |
| Biotin | -6.03 | 9.19E-03 | Down |
| Glutarylglycine | -6.17 | 2.12E-02 | Down |
| Uridine | -6.62 | 1.89E-02 | Down |
| Hydroxyhexanoycarnitine | -6.94 | 7.74E-03 | Down |
| Glutaric acid | -7.77 | 1.44E-02 | Down |
| D-Pantothenic Acid | -7.78 | 2.01E-02 | Down |
| Spermidine | -7.84 | 2.20E-02 | Down |
| Gamma-Glu-Leu | -7.85 | 8.93E-03 | Down |
| Hexanoylcarnitine | -8.03 | 8.78E-05 | Down |
| L-Asparagine | -8.31 | 9.59E-04 | Down |
| 3-Indoxylsulfate | -8.36 | 5.54E-03 | Down |
| Gln Ile Gln | -8.40 | 2.57E-02 | Down |
| Isovalerylglutamic acid | -8.70 | 1.10E-02 | Down |
| Leu Glu | -8.77 | 8.02E-04 | Down |
| 5-Fluorouridine | -9.52 | 3.96E-03 | Down |
| Ile Ile | -9.88 | 2.67E-02 | Down |
| B-Octylglucoside | -10.74 | 2.43E-02 | Down |
| Propionylcarnitine | -11.25 | 4.30E-03 | Down |

**References**

1. Aletaha D, Neogi T, Silman AJ, Funovits J, Felson DT, Bingham CO, 3rd, Birnbaum NS, Burmester GR, Bykerk VP, Cohen MD *et al*: **2010 Rheumatoid arthritis classification criteria: an American College of Rheumatology/European League Against Rheumatism collaborative initiative**. *Arthritis Rheum* 2010, **62**(9):2569-2581.

2. Arnett FC, Edworthy SM, Bloch DA, McShane DJ, Fries JF, Cooper NS, Healey LA, Kaplan SR, Liang MH, Luthra HS *et al*: **The American Rheumatism Association 1987 revised criteria for the classification of rheumatoid arthritis**. *Arthritis Rheum* 1988, **31**(3):315-324.

3. Liu T: **Chinese medicine industry standard of the People's Republic of China**. *Criteria for the diagnosis and treatment of diseases and syndromes in traditional Chinese medicine* 2006.

4. Tautenhahn R, Patti GJ, Rinehart D, Siuzdak G: **XCMS Online: a web-based platform to process untargeted metabolomic data**. *Anal Chem* 2012, **84**(11):5035-5039.

5. Chong J, Soufan O, Li C, Caraus I, Li S, Bourque G, Wishart DS, Xia J: **MetaboAnalyst 4.0: towards more transparent and integrative metabolomics analysis**. *Nucleic Acids Res* 2018, **46**(W1):W486-W494.

6. Szklarczyk D, Santos A, von Mering C, Jensen LJ, Bork P, Kuhn M: **STITCH 5: augmenting protein-chemical interaction networks with tissue and affinity data**. *Nucleic Acids Res* 2016, **44**(D1):D380-384.
